# Genetic Alteration, Prognostic and Immunological Role of Acyl-CoA Synthetase Long-Chain Family Member 4 in a Pan-Cancer Analysis

**DOI:** 10.3389/fgene.2022.812674

**Published:** 2022-01-20

**Authors:** Yongsheng Yu, Xuepu Sun, Fei Chen, Miao Liu

**Affiliations:** ^1^ Department of General Surgery, The Fourth Affiliated Hospital of Harbin Medical University, Harbin, China; ^2^ Department of General Surgery, The First Affiliated Hospital of Harbin Medical University, Harbin, China; ^3^ Department of General Surgery, Linyi Traditional Chinese Medicine Hospital, Linyi, China; ^4^ Department of Pathology, Beidahuang Industry Group General Hospital, Harbin, China

**Keywords:** ACSL4, Pan-cancer analysis, prognosis, immunological, genetic alteration

## Abstract

Acyl-CoA Synthetase long-chain family member 4 (ACSL4) is a member of acyl-CoA synthetase protein long-chain family, which is associated with amino acid synthesis, lipid synthesis and lipid peroxidation dependent iron death. However, the role of ACSL4 in generalized carcinoma remains unclear. We aim to analyze the expression and prognostic value of ACSL4 in pan-cancer, and further explore the correlation between ACSL4 and immune infiltration. Through ONCOMINE, TIMER (Tumor Immune Estimation Resource), GEPIA (Gene expression Profiling Interactive), UALCAN and HPA, ACSL4 expression patterns of in pan-cancer were analyzed. The prognostic value of ACSL4 was analyzed using PrognoScan and Kaplan-Meier Plotter databases. Furthermore, gene variation and epigenetic modification of ACSL4 were analyzed by cBioPortal and GSCA databases. Meanwhile, GEPIA and TIMER databases applied to evaluate the relationship between ACSL4 expression and immune infiltration. These results indicate that ACSL4 expression is down-regulated and associated with prognosis in most tumors. In general, lower ACSL4 expression shows more beneficial prognosis. The most common genetic alteration of ACSL4 is point mutation. ACSL4 is negatively correlated with DNA methylation levels in most cancers. ACSL4 mutations or hypomethylation are associated with poor prognosis. In addition, ACSL4 is positively correlated with immune infiltration in cancers. ACSL4 and immune infiltration are strongly associated with prognosis in BRCA (Breast invasive carcinoma) and SKCM (Skin Cutaneous Melanoma). ACSL4 mutation caused significant changes of immune infiltration in UCEC (Uterine Corpus Endometrial Carcinoma) and SARC (Sarcoma). ACSL4 may be a promising prognostic biomarker for pan-cancer and is closely associated with immune infiltration in the tumor microenvironment.

## Introduction

Cancer relies on metabolic reprogramming to drive malignant transformation (Boroughs and DeBerardinis, 2015). More and more tumor metabolic phenotypes have been identified and validated and are becoming biomarkers for the disease (Chen et al., 2019; Qin et al., 2020). Alterations in glucose and lipid metabolic pathways are one of the most striking metabolic features present in many different types of cancer. Changes in lipid metabolism of cancer cells showed increased proliferation, progression and metastasis (Cheng et al., 2018; Snaebjornsson et al., 2020). Activation of fatty acids catalyzed by acyl-coA synthase (ACS) is essential for the metabolism of both extracellular derived and *de novo* synthesized fatty acids. There are five members of the ACSL family, namely ACSL1, ACSL3, ACSL4, ACSL5 and ACSL6. Previous studies have shown that almost all ACSL members are deregulated in clinical cancers, which is conducive to excessive lipid biosynthesis and deposition, and ultimately makes the body susceptible to metabolic disorders and carcinogenesis (Tang et al., 2018). ACSL1 and ACSL4 are overexpressed in most cancer types and may have synergistic effects to promote unregulated cell growth, promote tumor invasion and evade programmed cell death (Sánchez-Martínez et al., 2017; Yen et al., 2017). While ACSL5 showed the opposite effect and was associated with a good prognosis in breast cancer patients. In addition, each with unique substrate of the ACSL family preferences and enzyme activity at different cellular sites, which depended on the chain length and saturation status of fatty acids (Grevengoed et al., 2014). ACSL4 is mainly present in peroxisome, mitochondria and endoplasmic reticulum. In contrast, ACSL4 prefers longer polyunsaturated fatty acids (PUFA) as substrates, such as arachidonic acid. It catalyzes the conversion of free arachidonic acid to arachidonic acid-coA ester, which is then esterified by interaction with membrane phospholipids and leads to ferroptosis (Küch et al., 2014; Kagan et al., 2017; Sánchez-Martínez et al., 2017). Recent studies have linked ferroptosis to a variety of cancers. Ferroptosis is a new iron-dependent form of regulated cell death,which characterized by iron-dependent accumulation of lipid-ROS and subsequent depletion of polyunsaturated fatty acid phospholipids (PUFA-PLS) (Hirschhorn and Stockwell, 2019). ACSL4 has been shown to be overexpressed in several cancer types, including colon cancer, breast cancer, liver and prostate cancer (Sánchez-Martínez et al., 2017; Wang J. et al., 2020; Chen et al., 2020; Ma et al., 2021; Sha et al., 2021), but often down-regulated in gastric cancer and lung cancer (Ye et al., 2016; Zhang et al., 2021).

As a percentage of genome length, the level of Copy number variation (CNV) in the tumor genome reflects the degree of tumor genome change (Hieronymus et al., 2018). The level of genomic copy number alteration is associated with prognosis in many cancers (Cao et al., 2019; Su et al., 2019; Lu et al., 2020; Woo et al., 2021). Recent analyses have linked cancer genomic characteristics, including TMB and CNA, to anti-tumor immunity. Studies suggest that high mutation load and low aneuploidy may be associated with increased T cell response (Budczies et al., 2018). The tumor microenvironment (TME) is composed of cellular components and non-cellular extracellular matrix. Cellular components include stromal fibroblasts, infiltrating immune cells, blood and lymphatic networks. TME is usually characterized by nutritional competition, low PH, hypoxia, and metabolite accumulation. This environment results in immunosuppression or a tolerant phenotype of immune cells and promotes metabolism that relies more on oxidative phosphorylation and fatty acid oxidation to meet energy requirements (Bader et al., 2020). The role of metabolic reprogramming in the activation and differentiation of immune cells has also attracted increasing attention (Biswas, 2015). As an important part of tumor environment, immune cell infiltration has been extensively studied for its role and prognostic value in various malignant tumors. With the development of immunotherapy, potential targets have been gradually discovered. But only a small percentage of patients with specific cancer types respond well to current immunotherapies. Therefore, it is necessary to explore the characteristics of various immune cells and their relationship with tumor interaction.

In this study, we analyzed the expression pattern and prognostic value of ACSL4 in various cancers using data from multiple public databases and discovered the impact of genetic changes of ACSL4 on prognosis. In addition, the potential relationship between ACSL4 expression and immune infiltration level was discussed. The results suggest that ACSL4 can affect the prognosis of cancer patients through its interaction with invasive immune cells.

## Materials and Methods

### Differential Gene Expression Analysis of ACSL4

We used Oncomine (www.oncomine.org), The TIMER (Tumor Immune Estimation Resource, https://cistrome.shinyapps.io/timer/) and GEPIA (Gene Expression Profiling Interactive Analysis, http://gepia.cancer-pku.cn/) to analyze ACSL4 expression in pan-cancer (Rhodes et al., 2004; Li et al., 2017; Tang et al., 2017). Specifically, the Oncomine Gene Expression Array Dataset is an online oncogene microarray database and data mining platform containing 65 gene expression datasets, including nearly 48 million gene expression measurements from more than 4,700 microarray experiments.Thresholds was determined based on the following values: *p* = 0.001, folding change 1.5, and gene ranking of all. TIMER is a visual and interactive site for comprehensive research on tumor immune interactions. It precalculated the levels of six tumor osmotic immune subsets for 10,897 tumors from 32 cancer types to explore associations between immune osmosis and various factors, including gene expression, clinical outcomes, somatic mutations, and somatic copy number changes. GEPIA is an interactive web-based application for gene expression analysis of 9736 tumors and 8587 normal samples based on TCGA and GTEx databases. It can carry out differential expression analysis, Survival analysis, Correlation analysis and similar gene detection analysis. The method for differential analysis is one-way ANOVA, | log2FC| = 1 and *p* = 0.01 is used as the cut-off standard. The Cancer Cell Line Encyclopedia (CCLE) (https://sites.broadinstitute.org/ccle/) is an effort to generate large-scale profiling data sets across nearly 1,000 cell lines from diverse tissue lineages. (Nusinow et al., 2020). DepMap Portal can be used for CCLE Data Visualization and Analysis. ACSL4 expression in cancer cell lines was validated by DepMap Portal.

The types of cancer acronyms analyzed in this study are as follows: ACC (adrenocortical carcinoma); BLCA (bladder urothelial carcinoma); BRCA (breast invasive carcinoma); CESC (cervical squamous cell carcinoma); CHOL (cholangiocarcinoma); COAD (colon adenocarcinoma); DLBC (lymphoid neoplasm diffuse large B cell lymphoma); ESCA (esophageal carcinoma); GBM (glioblastoma multiforme); HNSC (head and neck squamous cell carcinoma); KICH (kidney chromophobe); KIRC (kidney renal clear cell carcinoma); KIRP (kidney renal papillary cell carcinoma); LAML (lacute myeloid leukemia); LGG (brain lower grade glioma); LIHC (liver hepatocellular carcinoma); LUAD (lung adenocarcinoma); LUSC (lung squamous cell carcinoma); MESO (mesothelioma); OV (ovarian serous cystadenocarcinoma); PAAD (pancreatic adenocarcinoma); PCPG (pheochromocytoma and paraganglioma); PRAD (prostate adenocarcinoma); READ (rectum adenocarcinoma); SARC (sarcoma); SKCM (skin cutaneous melanoma); STAD (stomach adenocarcinoma); TGCT (testicular germ cell tumors); THCA (thyroid carcinoma); THYM (thymoma); UCEC (uterine corpus endometrial carcinoma); UCS (uterine carcinosarcoma); and UVM (uveal melanoma).

### Differentially Expressed at ACSL4 Protein Level

UALCAN (http://ualcan.path.uab.edu/analysis-prot.html) is used to analyze the cancer Omics data interactive Web resources (Chandrashekar et al., 2017). It analyzed protein expression using TCGA level 3 RNA-SEQ and clinical data from 31 cancer types. In this study, protein expression of ACSL4 between different cancer tissues and normal tissues was analyzed according to CPTAC workflow. *p* < 0.05 was considered to be significant. The HPA (Human Protein Atlas, www.proteinatlas.org) is a valuable tool for studying protein localization and expression in human tissues and cells, combining antibody-based approaches with transcriptional data. It has more than 10 million images showing patterns of protein expression at the single-cell level (Thul and Lindskog, 2018). In this study, immunohistochemical images of ACSL4 protein expression between normal and cancer tissues were observed by HPA.

### Analysis of ACSL4 Gene Variation

We used the cBio Cancer Genomics Portal (http://cbioportal.org) and GSCA (http://bioinfo.life.hust.edu.cn/GSCA/#/) to analyze variations in ACSL4 in different cancers, including mutations and copy number abnormalities (Cerami et al., 2012; Liu et al., 2018). UALCAN and MethSurv were used to analyze ACSL4 methylation and its correlation with survival prognosis in different cancers (Modhukur et al., 2018). Specifically, cBioPortal is an open platform for cancer genomics, providing data from more than 5,000 tumor samples from 20 cancer studies. The portal also includes copy number changes, changes in mRNA expression based on microarray and RNA sequencing, DNA methylation values, and protein and phosphoprotein levels. GSCA is provide a series of services to perform gene set genomic (Expression, SNV, CNV and methylation) and immunogenomic (24 immune cells) analyses. MethSurv is a survival analysis network tool based on CpG methylation pattern. It includes 7358 methylomes from 25 different human Bombs. Survival analysis of patient methylation levels at any CpG site (probe) was performed using the Cox proportional hazard model. Hazard ratio (HR) with 95% CI is derived from Cox fitting.

### Survival Prognosis Analysis of ACSL4

We use PrognoScan (http://www.abren.net/PrognoScan/), Kaplan-Meier (https://kmplot.com/analysis/) to analyzed the correlation between ACSL4 expression and survival was found in different carcinoma (Mizuno et al., 2009; Yuan et al., 2019). PrognoScan is a clinically annotated and extensive publicly available cancer microarray dataset for assessing the biological relationship between gene expression and prognosis. A univariate Cox *p* < 0.05 was defined as statistically significant. Kaplan-Meier is a powerful online tool that can be used to assess the effect of 54,000 genes on survival in 21 cancer types. We analyzed the relationship of ACSL4 expression with overall survival (OS) and relapse-free survival (RFS) in different cancer, Hazard ratios (HR) with 95% confidence intervals (CI) and log-rank P-values were calculated.

### Analysis of ACSL4 Expression and Immune Cell Infiltration Level

We used TIMER database to analyze the correlation between ACSL4 expression and the level of immune cell infiltration, including CD4^+^ T cells, CD8^+^ T cells, macrophages, neutrophils, monocytes, NK, DC, cancer-associated fibroblast (CAF) and Myeloid derived suppressor cell (MDSC). We used GSCA to analyze the correlation between single nucleotide variation (SNV), CNV levels in ACSL4 and immune cell infiltration levels in different cancers. Meanwhile, TIMER and GEPIA were used to analyze the correlation between ACSL4 and immune cell markers. Correlation analysis was determined by Spearman. *p* < 0.05 was considered statistically significant.

### ACSL4 Related Drugs Discovery in CMap

The Connectivity Map (https://clue.io/cmap), or CMap, is an open database that can be used to identify connections among small molecules which sharing a mechanism of chemicals, physiological processes and action, and then predict potential drugs in silicon (Wang C. Y. et al., 2020). The Touchstone module can be used to explore connectivities between signatures from ∼3,000 drugs and genetic loss/gain of function of ∼2,000 genes. Sets of compound perturbagens with enrichment scores above 90 (similar) and below -90 (opposing). We used CMap analysis tools to identify the association between ACSL4 and drugs.

## Results

### Differential Expression Analysis ACSL4 Between Tumor and Normal Samples

**FIGURE 1:**
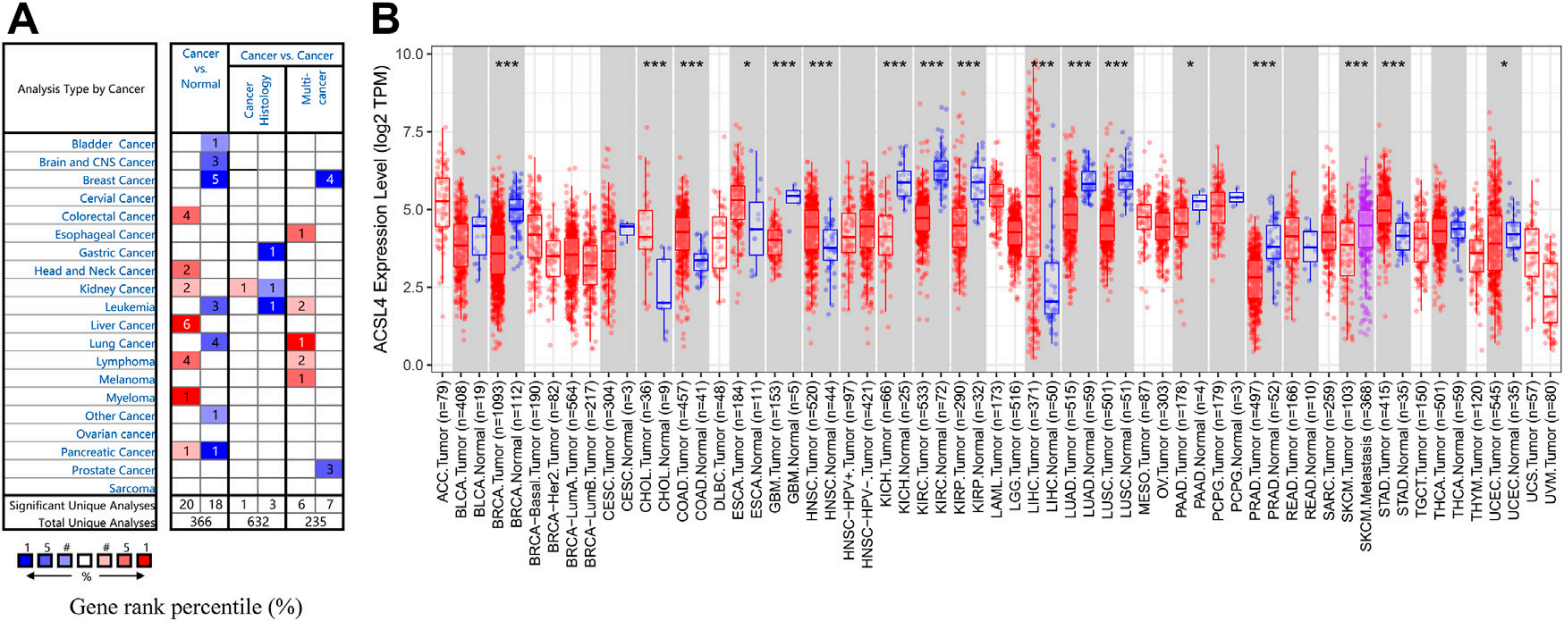
mRNA Expression Levels of ACSL4 in different Cancers. **(A)**. High or low expression of ACSL4 in different human cancer tissues compared with normal tissues in Oncomine database. The number in each cell present the amount of datasets. **(B)**. ACSL4 expression level in different cancers or specific cancer subtypes with corresponding normal tissues from the TCGA database in TIMER (*p < 0.05, **p < 0.01, ***p < 0.001).

To evaluate ACSL4 mRNA expression patterns in different cancers, we analyzed ACSL4 expression between normal tissues and tumors using Oncomine database (Figure 1A). The results showed that compared with normal tissues, ACSL4 expression was higher in colorectal cancer, head and neck cancer, kidney cancer, liver cancer, lymphoma, myeloma, and pancreatic cancer, but decreased in bladder cancer, brain and CNS cancer, breast cancer, leukemia, lung cancer, and pancreatic cancer. Notably, in the pancreatic cancer data set, one went up and the other went down. Further using the TIMER database to verify the differences between ACSL4 in different tumors and normal tissues (Figure 1B). The results showed that ACSL4 was significantly increased in CHOL, COAD, ESCA, LIHC, STAD and HNSC (*p* < 0.05). In addition, ACSL4 was Significantly decreased in BRCA Breast invasive carcinoma, KICH, KIRP, GBM, PAAD, SKCM, KIRC, PRAD and UCEC (*p* < 0.05). We also performed analysis using GEPIA and obtained consistent results (Supplementary Figure S1). By assembling the Cancer Cell Line Encyclopedia (CCLE), we further analyzed the expression of ACSL4 in cancer cell lines. The results showed that ACSL4 was highly dependent on leukemia, bladder cancer, lymphoma and lung cancer cell lines. The expression of ACSL was low in breast Cancer and prostate cancer cell lines, but high in liver cancer, thyroid cancer and skin cancer cell lines, which was consistent with the previous results (Supplementary Table S1).

**FIGURE 2:**
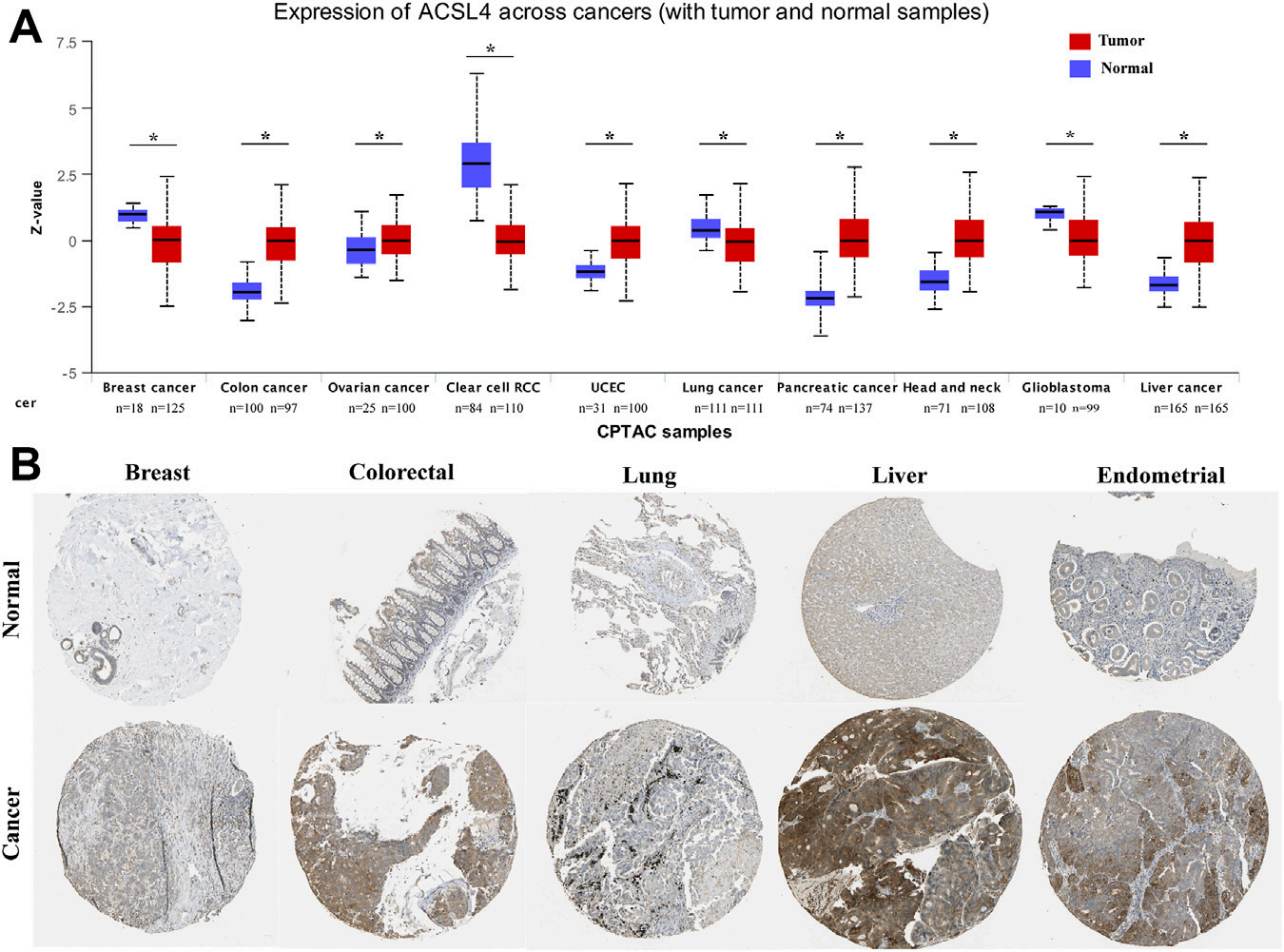
Protein expression levels of ACSL4 in different Cancers. **(A)**. The protein expression of ACSL4 in six cancers and normal tissues from the UALCAN database (*p < 0.05). Z-values represent standard deviations from the median across samples for the given cancer type. Log2 Spectral count ratio values from CPTAC were first normalized within each sample profile, then normalized across samples. UCEC (uterine corpus endometrial carcinoma). **(B)**. the protein expression of ACSL4 in tumors and normal tissues were obtained from the Human Protein Atlas.

In order to determine the protein expression of ACSL4 in various cancers, the UALCAN database was used to analyze the ACSL4 protein expression. The results showed that compared with normal tissues, ACSL4 expression was higher in COAD, OV and UCEC tissues (*p* < 0.05), but lower in BRAC and KIRC tissues (*p* < 0.05, Figure 2A). This was consistent with ACSL4 mRNA expression in TIMER and GEPIA databases. Meanwhile, compared with normal tissues, ACSL4 was strongly positive in endometrial cancer and liver cancer, and low in breast, lung, and renal cell carcinoma tissues in the human protein atlas (Figure 2B).

### Prognostic Analysis of ACSL4 in Patients with Different Cancers

**FIGURE 3:**
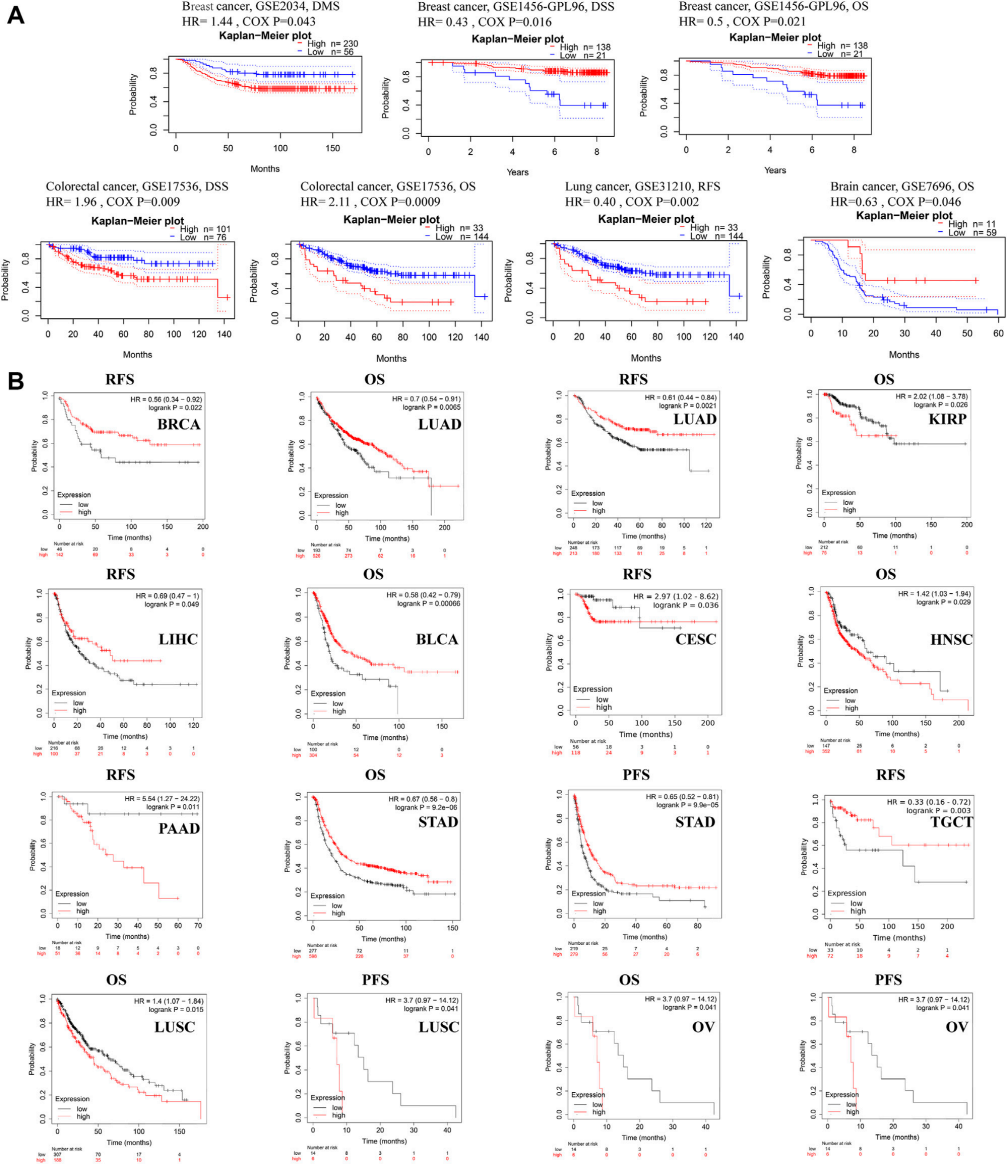
Survival data evaluating the prognostic value of ACSL4. **(A)**. Correlation between ACSL4 and prognosis of various types of cancer in the PrognoScan. DMFS (*n* = 286) in breast cancer cohort GSE 2034. DSS (n = 159) in breast cancer cohort GSE1456-GPL96. OS (*n* = 159) in breast cancer cohort GSE1456-GPL96. DSS (*n* = 177) in colorectal cancer cohort GSE17536. OS (*n* = 177) in colorectal cancer cohort GSE17536. RFS (*n* = 204) in lung cancer (Adenocarcinoma) cohort GSE31210. OS (*n* = 70) in brain cancer (Glioblastoma) cohort GSE7696. DMFS, distant metastasis-free survival; OS, overall survival; DSS, disease-specific survival; RFS, relapse-free survival. **(B)**. Correlation between ACSL4 and prognosis of various types of cancer in the Kaplan-Meier plotter database. RFS of BRCA (breast invasive carcinoma), OS and RFS of LUAD (lung adenocarcinoma); OS of KIRP (kidney renal papillary cell carcinoma); RSF of LIHC (liver hepatocellular carcinoma); OS of BLAC (bladder urothelial carcinoma); RFS of CESC (cervical squamous cell carcinoma); OS of HNSC (head and neck squamous cell carcinoma); RFS of PAAD (pancreatic adenocarcinoma); OS and PFS of STAD (stomach adenocarcinoma); RFS of TGCT (testicular germ cell tumors); OS and PFS of LUSC (lung squamous cell carcinoma); OS and PFS of OV (ovarian serous cystadenocarcinoma). Red curve represents patients with high expression of ACSL4. OS, overall survival; PFS, Progression-free survival; RFS, recurrence-free survival.

The relationship between ACSL4 expression and prognosis in patients with various cancers was analyzed using the PrognoScan database (Figure 3A). We found that ACSL4 was associated with prognosis of four cancer types. The patients with high expression of ACSL4 have poor prognosis in colorectal cancer (OS: total number, 177; 95% CI, 1.36–3.28, HR, 2.11; Cox *P*, 0.000896,433; DSS: total number, 177; 95% CI, 1.19–3.25, HR, 1.96; Cox *P*, 0.00877,945). While low expression of ACSL4 has poor prognosis in lung cancer (RFS: total number, 204; 95% CI, 0.22–0.71, HR, 0.40; Cox *P*, 0.008784533), brain cancer (OS: total number, 70; 95% CI, 0.41–0.99, HR, 0.63.11; Cox *P*, 0.0457,155) and breast cancer (DSS: total number, 159; 95% CI, 0.22–0.85, HR, 0.43; Cox *P*, 0.0158,694; OS: total number, 159; 95% CI, 0.28–0.90, HR, 0.50; Cox *P*, 0.0205,638). However, another data set of breast cancer showed high expression of ACSL4 and poor prognosis (DMFS: total number, 286; 95% CI, 1.01–2.05, HR, 1.44; Cox *P*, 0.0432,055). The same analysis was performed on the Kaplan-Meier mapping database and ACSL4 expression was associated with prognosis for 14 cancer types (Figure 3B). Brain cancer was not analyzed in kaplan-Meie database. Compared with PrognoScan database, low ACSL4 expression was associated with poorer prognosis in LUAD (OS: HR = 0.7; 95% CI, 0.54–0.91; Cox *p* = 0.0065; RFS: HR = 0.61; 95% CI, 0.44–0.84; logrank *p* = 0.0021), BRCA (RFS: HR = 0.56; 95% CI, 0.34–0.92; logrank *p* = 0.022). There was no significant association in rectal adenocarcinoma (OS: HR = 1.91; 95% CI, 0.8–0.455; logrank *p* = 0.14; RFS: HR = 3.4; 95% CI, 0.39–29.73; logrank *p* = 0.24). In addition, high ACSL4 expression in KIRP and LIHC has a poor prognosis. Low ACSL4 expression was associated with poor prognosis in six other cancers, including BLCA, CESC, HNSC, PAAD, STAD and TGCT. The prognostic analysis of ACSL4 in OV (OS: HR = 1.16; 95% CI, 1.01–1.33; logrank *p* = 0.03; RFS: HR = 0.83; 95% CI,0.7–0.98; logrank *p* = 0.031) and LUSC (OS: HR = 1.4; 95% CI, 1.07–1.84; logrank *p* = 0.015; RFS: HR = 3.7; 95% CI, 0.97–14.12; logrank *p* = 0.041) showed opposite trends. Further use of the GEPIA database to assess the correlation between ACSL4 expression and patient outcome was consistent with the PrognoScan and Kaplan-Meier results. Patients with high ACSL4 expression in CHOL, LIHC and LUAD had poor prognosis; patients with low ACSL4 expression in KIRC, ACC, LGG, PCPG and SKCM had poor prognosis (Supplementary Figure S2). In summary, the combined analysis of the three databases shows that ACSL4 has prognostic value in certain cancers, which may be beneficial or harmful. In general, low ACSL4 expression has shown a beneficial role in pan-cancer.

### Genetic Alterations of ACSL4 in Different Cancers

**FIGURE 4:**
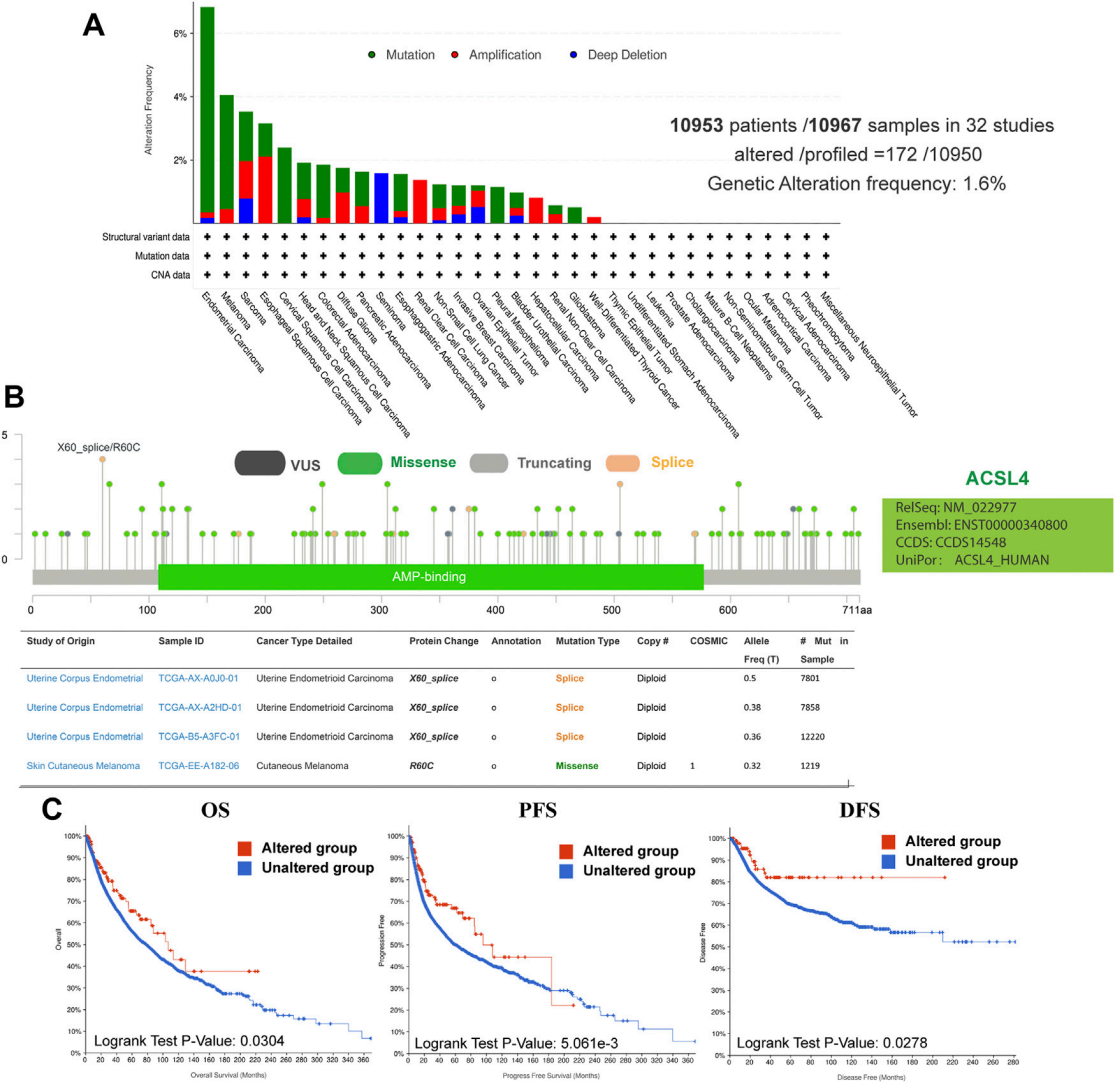
Genetic alterations of ACSL4 in different cancers from the cBioPortal database. **(A)**. Genetic aberration of ACSL4 in tumors using cBioPortal-TCGA pan-cancer panel. This data includes 10953 patients/10967 samples in 32 studies. The global genetic alteration frequency is 1.6%. **(B)**. The types and mutation sites of ACSL4 gene. **(C)**. Correlation between ACSL4 altered and prognosis of various types of cancer. OS of pan-cancer in 32 studies (*n* = 10803); PFS of pan-cancer (*n* = 10613) in 32 studies; DFS of pan-cancer (*n* = 5383) in 32 studies. OS, overall survival; PFS, Progression-free survival; DFS, Disease-free survival.

Using the cBioPortal database, we explored the genetic changes of ACSL4 in various cancers and their correlation with patients’ OS and PFS in TCGA-Pan cancer panel. In 10,953 patients with ACSL4 mutation information from the TCGA dataset, the percentage of ACSL4 mutation was 1.6% (Figure 4A). The most common mutations are point mutations, which are mainly found in endometrial cancer, melanoma, cervical squamore cell carcinoma, pleural mesothelioma and Glioblastoma. All cases of Melanoma have point mutations. “Amplification” exists in all cases of Renal clear cell carcinoma, Hepatocellular carcinoma and well-differentiated thyroid cancer. The types and mutation sites of ACSL4 gene are shown in the figure (Figure 4B). Among mutations, missense mutation has the highest frequency. Critical to changes in ACSL4 function are mutations in AMP-biding containing the active site of ACSL4. The most common site was X60-splice/R60C, which was found in 3 cases of endometrial carcinoma and 1 case of cutaneous melanoma. We explored the potential relationship between genetic changes in ACSL4 and clinical outcomes in patients with different types of cancer. The results showed that the OS (*p* = 0.034), DFS (*p* = 0.0278) and PFS (*p* = 0.005) of patients with ACSL4 mutation were significantly higher than those without ACSL4 mutation (Figure 4C). In short, genetic alterations in ACSL4 can significantly affect patient outcomes. Analysis of CCLE database showed that “copy number amplification” occurred frequently in cervical cancer, fibroblast, ovarian cancer, breast cancer and endometrial/uterine cancer. The most frequently mutated entity is lymphoma, followed by Leukemia. In addition, endometrial/uterine cancer and lung cancer mutation frequency is high, which is consistent with cBioPortal results (Supplementary Table S1).

**FIGURE 5:**
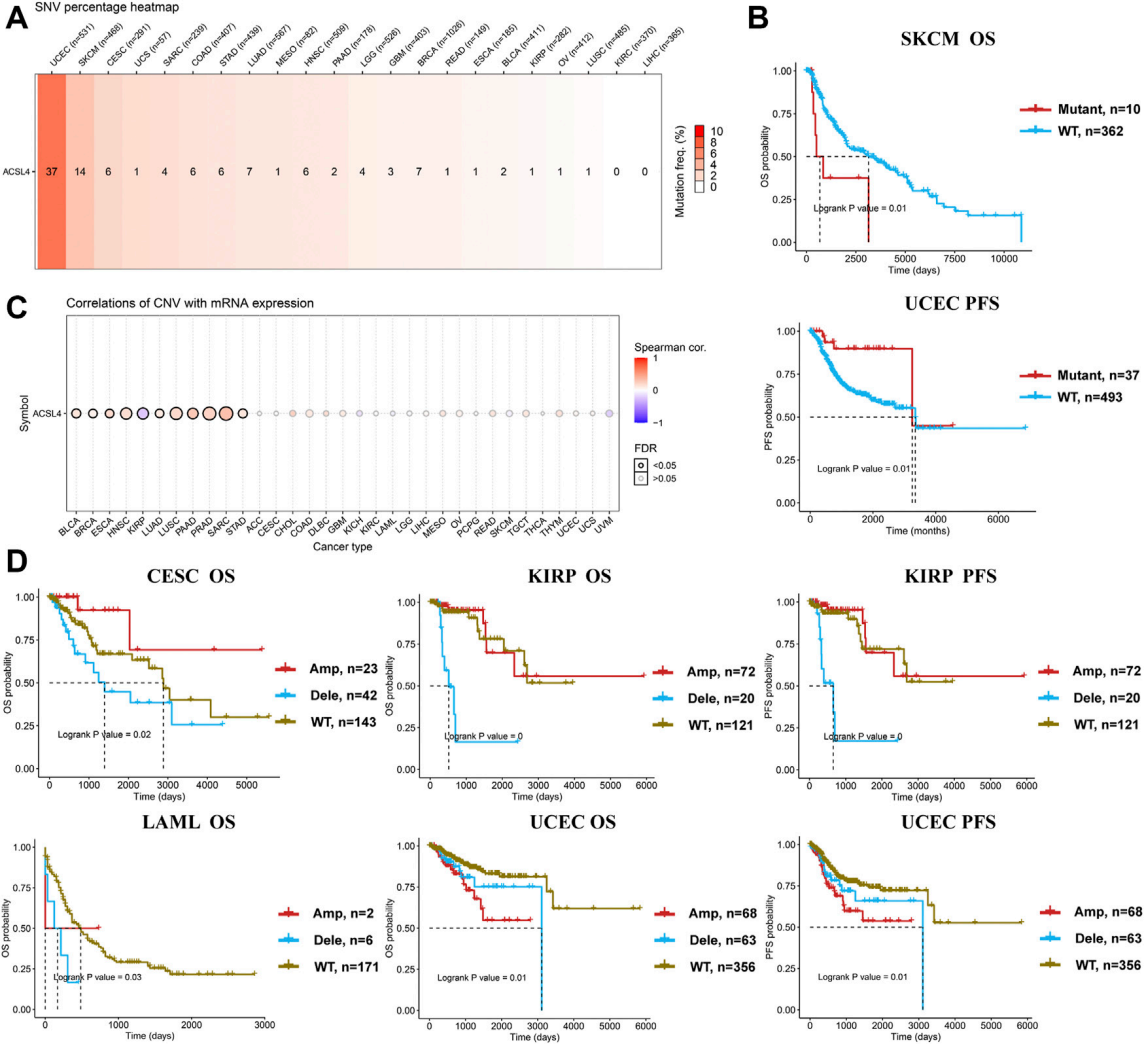
Genetic alterations of ACSL4 in different cancers from the GSCA database. **(A)**. SNV percentage heatmap of ACSL4 in different cancers. **(B)**. Correlation between ACSL4 mutant and prognosis in SKCM and UCEC. OS of SKCM (skin cutaneous melanoma); PFS of UCEC ((uterine corpus endometrial carcinoma). OS, overall survival; PFS, Progression-free survival. **(C)**. Correlation of CNV with mRNA expression in different cancers. **(D)**. Correlation between ACSL4 CNV and prognosis in different cancers. OS of CESC (cervical squamous cell carcinoma); OS and PFS of KIRP (kidney renal papillary cell carcinoma); OS of LAML (lacute myeloid leukemia); OS and PFS of UCEC (uterine corpus endometrial carcinoma). OS, overall survival; PFS, Progression-free survival.

Meanwhile, GSCA database was used to analyze the SNV of ACSL4, and SNV was found in 19 kinds of cancer (Figure 5A). Consistent with cBioPortal, mutations were most common in UCEC and SKCM. Moreover, patients with ACSL4 mutations had a shorter overall survival time than those with wild-type in SKCM (HR, 2.76, Cox *p* = 0.01), while had a longer PFS than those with wild-type in UCEC (HR, 0.29, Cox *p* = 0.01) (Figure 5B). CNA is a common genetic alteration associated with the occurrence and progression of cancer by regulating the expression of tumor-related genes (Davoli et al., 2017). We analyzed the CNV of ACSL4 and the correlation between CNV and mRNA expression through GSCA database (Figure 5C). The results showed that ACSL4 CNV was positively correlated with mRNA expression in 10 cancers, including BLCA, BRCA, ESCA, HNSC, LUAD, LUSC, PAAD, PRAD, SARC and STAD. Only in KIRP, ACSL4 CNV was negatively correlated with mRNA expression. Similarly, we assessed the effect of ACSL4 CNV on prognosis in patients with various cancers. In CSEC, KIRP, and LAML, patients with copy number deletion had a worse prognosis, while patients with copy number amplification had a worse prognosis in UCEC (Figure 5D).

**FIGURE 6:**
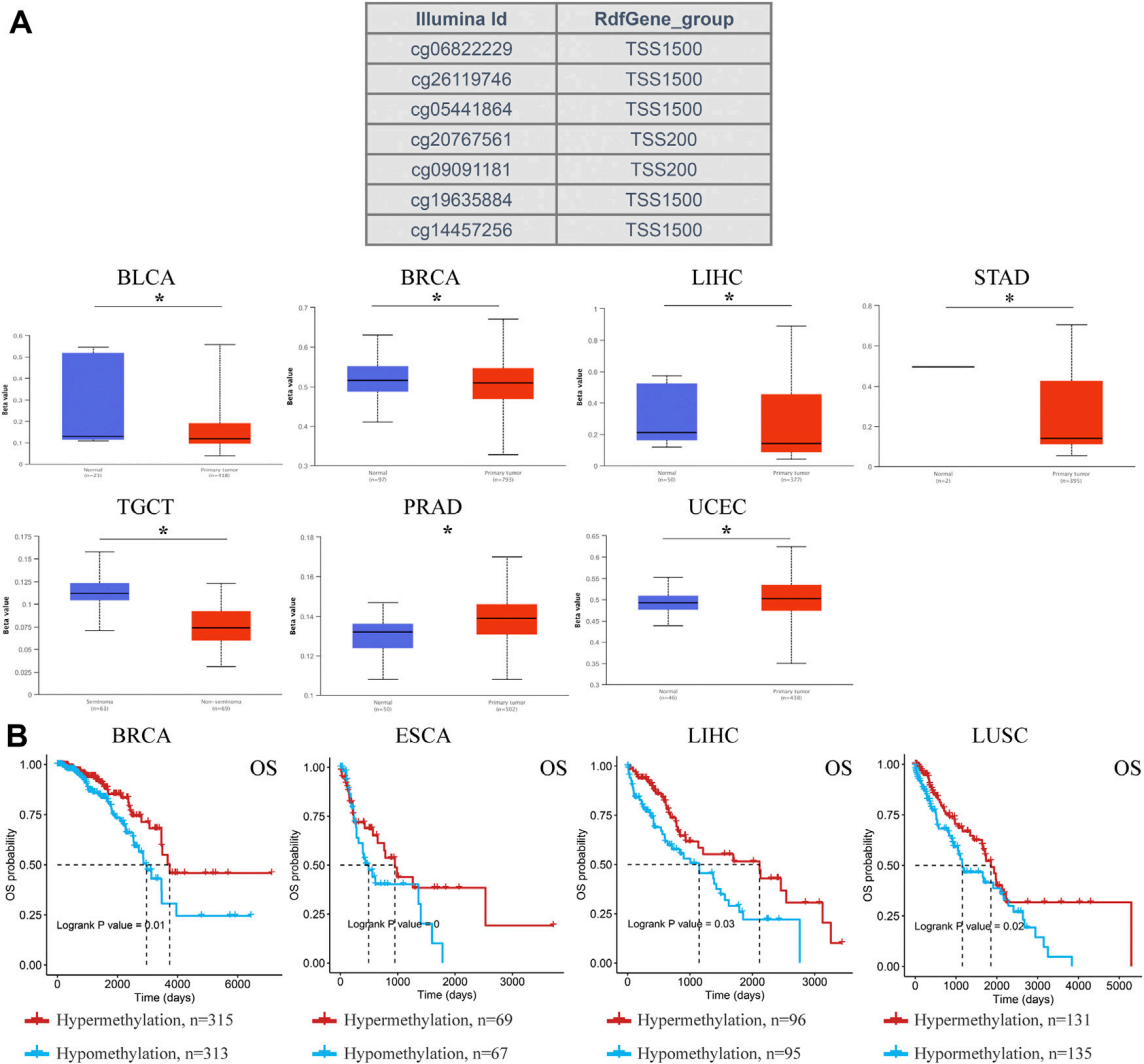
DNA methylation aberration of ACSL4 in different cancers from the UACLAN and GSCA database. **(A)**. Probes for detecting DNA methylation of ACSL4 promoter and ACSL4 promoter methylation profile in different cancers. ACSL4 hypomethylated in BLCA, BRCA, LIHC, STAD and TCGT, while higher methylation levels in PRAD and UCEC from UACLAN database (*p < 0.05). The Beta value indicates level of DNA methylation ranging from 0 (unmethylated) to 1 (fully methylated). Different beta value cut-off has been considered to indicate hyper-methylation (Beta value: 0.7–0.5) or hypo-methylation (Beta-value: 0.3–0.25). BLCA (bladder urothelial carcinoma); BRCA (breast invasive carcinoma); LIHC (liver hepatocellular carcinoma); STAD (stomach adenocarcinoma); TGCT (testicular germ cell tumors); PRAD (prostate adenocarcinoma); UCEC (uterine corpus endometrial carcinoma). **(B)**. Correlation between ACSL4 DNA hypomethylation and prognosis in different cancers from GSCA database. OS of BRCA (breast invasive carcinoma), ESCA (esophageal carcinoma), LIHC (liver hepatocellular carcinoma) and LUSC (lung squamous cell carcinoma). OS, overall survival.

DNA methylation regulates gene expression and closure and is closely related to human development and tumorigenesis. Subsequently, ACSL4 methylation in different cancers was analyzed using the UALCAN database (Figure 6A). The results showed that ACSL4 was hypomethylated in BLCA, BRCA, LIHC, STAD and TCGT. We found that lowly ACSL4 express tumors presented with decreased DNA methylation level of ACSL4, including BLCA and BRCA. In addition, DNA methylation of ACSL4 had higher levels in PRAD and UCEC. Similar results were obtained in GSCA database. ACSL4 was hypomethylated in LIHC and THCA, while it was hypermethylated in PRAD (Supplementary Figure S3A). Contrary to the UALCAN database results, ACSL4 was hypomethylated in UCEC. We further analyzed the correlation between ACSL4 methylation level and mRNA expression. The results showed that ACSL4 methylation was inversely correlated with mRNA in most cancers, including BRCA, CESC, COAD, ESCA, GBM, HNSC, KIRP, KIRC, LAML, LIHC, PCPG, PRAD, PEAD, SARC, SKCM, STAD, TGCT, THCA and UCEC. In BRCA, LIHC, LUSC, and ESCA (Supplementary Figure S3B). ACSL4 methylation levels were associated with poor prognosis (Figure 6B). The DNA methylation level of ACSL4 and the prognostic value of each single CpG were analyzed by MethSurv database (Supplementary Figure S3C). Compared with patients with ACSL4 hypermethylation, Hypomethylation was associated with poorer prognosis in 11 cancers, including BRCA, CESC, COAD, ESCA, GBM, KIRP, LGG, LIHC, STAD, SKCM and UCEC. Which is located in the CPG island site prognosis is poorer, including cg0544164, cg06822229, cg10721440 (Supplementary Figure S4). Patients with ACSL4 hypermethylation had poorer prognosis and survival in other cancers: ACC, HNSC, KIRC, LUSC, PAAD and UCS.

### Analysis of Immune Infiltration of ACSL4 in Different Cancers

**FIGURE 7:**
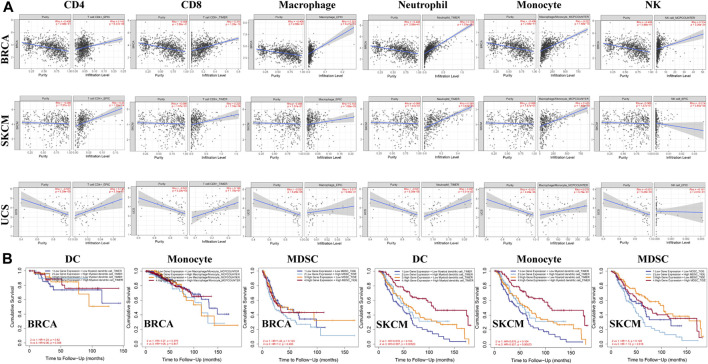
Analysis of immune infiltration of ACSL4 in different cancers from the TIMER database. **(A)**. ACSL4 expression has significantly negatively correlated with tumor purity and significantly positively correlated with the infiltration levels of CD4+T cell, CD8+T cell, macrophage, neutrophil, monocyte and NK in BRCA (breast invasive carcinoma). ACSL4 expression has no relation with tumor purity and significantly negatively correlated with NK, and significantly positively correlated with the infiltration levels of CD4+T cell, CD8+T cell, macrophage, neutrophil, monocyte and in SKCM (skin cutaneous melanoma). ACSL4 expression has significantly negatively correlated with tumor purity and NK, and no relation with infiltrating levels of CD4+T cell, CD8+T cell, macrophage, neutrophil, monocyte in UCS (uterine carcinosarcoma). *p* < 0.05 is considered as significant. **(B)**. Correlation between ACSL4 mRNA expression, with infiltrating levels of DC, monocyte, MDSC (Myeloid derived suppressor cell) and prognosis in BRCA (breast invasive carcinoma) and SKCM (skin cutaneous melanoma).

Studies have shown that tumor-infiltrating lymphocytes are associated with cancer survival. The correlation between ACSL4 expression and TIL level was analyzed by TISIDB database. The heat map showed that ACSL4 was positively correlated with most immune cells (Supplementary Figure S5). We also evaluated the association between ACSL4 expression and tumor-infiltrating immune cells in the TIMER database (Figure 7A). The results showed that ACSL4 expression was significantly associated with NK, B cells, macrophages, DC, CD4^+^ T cells, CD8^+^ T cells, monocytes and neutrophil infiltration in 13, 16, 19, 24, 25, 26, 28 and 31 cancer types. ACSL4 was positively correlated with neutrophils, CD4^+^ T cells and monocyte infiltration. There was no correlation between ACSL4 expression and immune cell infiltration in UCS. In addition, we also found that the cancer with high ACSL4 expression was positively correlated with the infiltration level of MDSC, including COAD, LIHC and STAD, whereas the cancer with low ACSL4 expression was negatively correlated with the infiltration level of MDSC, including BRCA, KIRC, LUSC, GBM and SKCM.

We further analyzed the effects of ACSL4 and immune cell infiltration levels on survival outcomes. The results showed that MDSC infiltration level was correlated with the prognosis of 16 types of Cancer patients, while neutrophils, cancer-associated fibroblast (CAF) and DC were correlated with the prognosis of 10, nine and nine types of Cancer patients, respectively. And in BRCA and SKCM, ACSL4 and the level of immune cell infiltration are closely related to survival prognosis (Figure 7B). These results indicate that ACSL4 plays an important role in regulating the immune cells infiltration in BRCA and SKCM, especially monocytes, DC and MDSC.

In addition, we analyzed the effect of ACSL4 mutation on immune cell levels in various cancers by Mutation module. The results showed that ACSL4 mutation caused significant changes in the levels of various immune cells, especially in UCEC and SARC. Compared with ACSL4 wild type, ACSL4 mutation caused significant changes in CD4^+^ T cells, DC, macrophages and CAF, and showed opposite trends in both types of cancer (Supplementary Figure S6A). Similarly, the SCAN module was used to analyze the influence of ACSL4 on the level of immune cell invasion in High Amplification or Deep Deletion. The results showed that abnormality of ACSL4 at different copy numbers mainly caused changes in CAF levels, including HNSC, LGG, OV and BRCA (Supplementary Figure S6B). In addition, it can also cause changes in neutrophils, CD8^+^ T cells, DC, NK and Tfh infiltration levels.

### Analysis of Immunoinfiltrating Cell Markers of ACSL4 in Various Cancers

Next, we analyzed the correlation between ACSL4 and immunoinfiltrating cell markers through TIMER and GEPIA databases respectively. We selected cancer types, mainly BRCA and SKCM, that express poor prognosis in cancers and are related to the level of immune cell infiltration and affect prognosis. UCS as a control. Immune cells include B cells, T cells (general), CD8^+^ T cells, Tfh, Treg, Exhaust T cells, TAM, M1 and M2 macrophages, monocytes, NK, neutrophils, DC, CAF and MDSC. After adjusting for tumor purity, ACSL4 expression was strongly associated with 57 of 58 BRCA immune cell markers, 41 of SKCM, and 24 of LIHC. In contrast, only three markers in UCS were significantly associated with ACSL4 (Table 1). Markers of CD8^+^ T cells, TAM, M2 macrophages, monocytes, DC, CAF and MDSC were most closely correlated with ACSL4 expression in BRCA and SKCM, while no correlation was found in USC. ACSL4 was significantly correlated with CCL2, CD68, IL10 of TAM, CD113, MS4A4A, VSIG4 of M2 macrophages, and CD86 and CSF1R of monocytes in BCRC and SKCM.

**TABLE 1:** Correlations between ACSL4 and gene markers of immune cells in TIMER.

Cell type	Gene markers	BRCA				SKCM				UCS			
		None		Purity		None		Purity		None		Purity	
		Cor	*p*	Cor	*p*	Cor	p	Cor	*p*	Cor	*p*	Cor	*p*
B	CD19	0.275	***	0.096	*	0.122	*	0.093	0.047	0.148	0.271	−0.046	0.746
	CD79A	0.324	***	0.138	***	0.087	0.087	0.05	0.282	−0.03	0.826	−0.179	0.2
T	CD2	0.457	***	0.314	***	0.23	***	0.23	***	0.134	0.322	−0.136	0.331
	CD3D	0.404	***	0.241	***	0.161	**	0.138	*	0.003	0.982	−0.259	0.061
	CD3E	0.436	***	0.278	***	0.131	*	0.097	0.039	−0.081	0.55	−0.274	0.047
CD8^+^ T cell	CD8A	0.444	***	0.314	***	0.214	***	0.204	***	0.196	0.144	0.029	0.838
	CD8B	0.405	***	0.28	***	0.157	**	0.132	*	0.069	0.612	0.043	0.759
Tfh	BCL6	0.259	***	0.244	***	0.388	***	0.378	***	0.241	0.071	0.136	0.333
	CXCR5	0.331	***	0.155	***	0.12	*	0.091	0.053	−0.051	0.709	−0.264	0.056
	ICOS	0.483	***	0.377	***	0.327	***	0.333	***	0.299	0.024	0.194	0.164
Treg	CCR8	0.5	***	0.444	***	0.358	***	0.445	***	0.35	*	0.191	0.172
	FOXP3	0.443	***	0.34	***	0.082	0.115	0.096	0.041	0.317	0.016	0.193	0.165
	IL2RA	0.583	***	0.507	***	0.231	***	0.435	***	0.438	**	0.299	0.029
	IL7R	0.673	***	0.6	***	0.354	***	0.51	***	0.502	***	0.388	*
Exhaust T	CTLA4	0.432	***	0.313	***	0.336	***	0.276	***	0.083	0.54	−0.065	0.644
	GZMB	0.41	***	0.295	***	−0.05	0.115	0.107	0.023	0.147	0.275	−0.103	0.461
	HAVCR2	0.578	***	0.509	***	0.208	***	0.334	***	0.326	0.013	0.142	0.309
	LAG3	0.246	***	0.155	***	0.118	0.023	0.122	*	−0.154	0.253	−0.391	*
	PDCD1	0.333	***	0.176	***	0.319	***	0.097	0.039	0.034	0.805	−0.244	0.079
TAM	CCL2	0.514	***	0.422	***	0.091	0.079	0.244	***	0.122	0.368	−0.001	0.996
	CD68	0.529	***	0.462	***	0.189	**	0.17	**	0.372	*	0.145	0.3
	IL10	0.543	***	0.459	***	0.167	*	0.387	***	0.158	0.24	−0.006	0.967
M1	IRF5	0.265	***	0.208	***	0.177	**	0.176	**	0.289	0.029	0.259	0.062
	NOS2	0.284	***	0.28	***	-0.058	0.264	-0.08	0.087	−0.178	0.185	0.018	0.901
	PTGS2	0.578	***	0.51	***	0.199	**	0.267	***	0.179	0.183	0.164	0.242
M2	CD163	0.557	***	0.5	***	0.03	0.567	0.433	***	0.329	0.013	0.216	0.12
	MS4A4A	0.572	***	0.492	***	0.091	0.082	0.373	***	0.286	0.031	0.137	0.328
	VSIG4	0.462	***	0.385	***	0.049	0.346	0.311	***	0.302	0.023	0.169	0.226
monocyte	CD86	0.595	***	0.525	***	0.179	**	0.406	***	0.358	*	0.157	0.261
	CSF1R	0.566	***	0.47	***	0.11	0.035	0.338	***	0.306	0.02	0.11	0.431
NK	NCAM1	0.445	***	0.359	***	0.043	0.414	0.153	*	−0.162	0.228	−0.03	0.834
	KIR2DL1	0.266	***	0.186	***	-0.092	0.075	0.041	0.381	−0.023	0.864	−0.008	0.954
	KIR2DL3	0.252	***	0.165	***	-0.018	0.726	0.021	0.658	0.203	0.13	0.073	0.604
	KIR2DL4	0.301	***	0.222	***	-0.062	0.237	0.086	0.068	0.052	0.7	−0.135	0.335
	KIR2DS4	0.237	***	0.237	***	-0.072	0.168	0.034	0.463	−0.006	0.962	−0.006	0.962
	KIR3DL1	0.334	***	0.334	***	-0.101	0.052	0.052	0.258	0.221	0.099	0.221	0.099
	KIR3DL2	0.296	***	0.296	***	0.085	0.101	0.1	0.031	0.105	0.438	0.105	0.438
	KIR3DL3	0.176	***	0.176	***	0.072	0.168	-0.039	0.394	0.138	0.306	0.138	0.306
	KLRD1	0.581	***	0.49	***	-0.051	0.327	0.328	***	0.39	*	0.187	0.179
	KLRK1	0.424	***	0.275	***	0.134	*	0.288	***	0.115	0.392	−0.086	0.541
Neutrophil	CCR7	0.365	***	0.199	***	0.313	***	0.123	*	0.17	0.205	0.009	0.947
	CEACAM8	0.042	0.166	0.075	0.018	0.034	0.514	0.063	0.178	−0.023	0.868	−0.021	0.88
	FUT4	0.654	***	0.586	***	0.308	***	0.42	***	0.217	0.105	0.209	0.133
	ITGAM	0.446	***	0.357	***	0.064	0.221	0.352	***	0.292	0.028	0.15	0.282
	MPO	0.356	***	0.286	***	0.066	0.206	0.056	0.228	−0.097	0.471	−0.04	0.778
DC	ITGAX	0.496	***	0.4	***	0.275	***	0.213	***	0.355	*	0.183	0.191
	CD1C	0.386	***	0.232	***	0.281	***	0.154	**	0.14	0.298	0.062	0.661
	HLA-DPA1	0.504	***	0.387	***	0.127	0.014	0.216	***	0.252	0.059	0.033	0.814
	HLA-DPB1	0.395	***	0.225	***	0.108	0.038	0.2	***	0.265	0.046	0.061	0.666
	HLA-DQB1	0.334	***	0.197	***	0.305	***	0.156	**	0.317	0.016	0.123	0.379
	HLA-DRA	0.534	***	0.425	***	0.118	0.023	0.304	***	0.398	*	0.173	0.215
	NRP1	0.656	***	0.603	***	0.031	0.551	0.485	***	0.45	**	0.302	0.028
CAF	HGF	0.536	***	0.456	***	0.193	**	0.431	***	−0.201	0.133	−0.206	0.14
	PDGFRB	0.478	***	0.39	***	0.35	***	0.122	*	0.01	0.94	−0.03	0.832
	TGFB1	0.343	***	0.206	***	0.289	***	0.191	***	0.378	0.004	0.221	0.112
	THBS1	0.465	***	0.454	***	0.314	***	0.392	***	0.457	**	0.406	*
MDSC	CD14	0.368	***	0.265	***	-0.029	0.581	0.186	***	0.229	0.086	0.088	0.529
	CD33	0.503	***	0.404	***	0.134	*	0.324	***	0.212	0.113	0.092	0.512

BRCA, breast invasive carcinoma; SKCM, skin cutaneous melanoma; UCS, uterine carcinosarcoma.

CAF, cancer-associated fibroblast; DC, dendritic cell; MDSC, myeloid derived suppressor cell; NK, natural killer cell; Tfh, follicular helper T cell; Th, T helper cell; Treg, regulatory T cell; TAM, tumor-associated- macrophage.

None, correlation without adjustment; Purity, correlation adjusted for tumor purity; Cor, R value of Spearman’s correlation. *p < 0.01; **p < 0.001; ***p < 0.0001.

In BRCA and SKCM, DC markers (CD1C, GAX, HLA-DPA1, HLA-DPB1, HLA-DPB2, HLA-DRA and NRP1) were significantly correlated with ACSL4 expression levels (Table 1). This supports that ACSL4 expression is closely related to DC infiltration, thus affecting the prognosis and survival of patients. Similarly, CAF markers (HGF, PDGFRB, FGB1, THBS1) and MDSC markers (CD14, CD33) were positively correlated with ACSL4 expression in BRCA and SKCM. These results suggest that ACSL4 plays a role in the immunosuppressive state of cancer. In order to verify the results of TIMER analysis, GEPIA database was further used to analyze the correlation between ACSL4 expression and immune cell markers (Table 2). The results were consistent with the TIMER database. In BRCA and SKCM, ACSL4 expression was significantly correlated with CD8^+^ T cells, TAM, M2 macrophages, monocytes, CAF and MDSC markers. There was no correlation between ACSL4 and DC markers CD1C and HLA-DQB1 in SKCM. Other markers were correlated with both cancers.

**TABLE 2:** Correlations between ACSL4 and Gene Markers of CD8^+^ T cell, TAM, M2, Neutrophil, CAF in GEPIA.

Cell type	Gene markers	BRCA		SKCM		UCS	
		R	*p*	R	*p*	R	*p*
CD8+T cell	CD8A	0.21	***	0.16	***	0.024	0.86
	CD8B	0.15	***	0.13	*	−0.022	0.87
TAM	CCL2	0.48	***	0.14	*	0.012	0.93
	IL10	0.45	***	0.25	***	0.068	0.61
	CD68	0.39	***	0.14	*	0.31	0.02
M2	CD163	0.34	***	0.31	***	0.089	0.51
	VSIG4	0.33	***	0.25	***	0.2	0.13
	MS4A4A	0.43	***	0.32	***	0.7	0.21
Neutrophil	CD86	0.44	***	0.33	***	0.21	0.12
	CSF1R	0.43	***	0.3	***	0.2	0.14
CAF	HGF	0.25	***	0.13	*	0.043	0.75
	PDGFRB	0.25	***	0.16	**	−0.05	0.71
	TGFB1	0.15	***	0.27	***	0.24	0.074
	THBS1	0.28	***	0.21	***	0.18	0.19
CAF	CD14	0.18	***	0.21	***	0.17	0.2
	CD33	0.33	***	0.22	***	0.15	0.26
DC	CD1C	0.22	***	0.025	0.59	0.062	0.65
	HLA-DPB1	0.23	***	0.19	***	0.1	0.45
	HLA-DQB1	0.13	***	0.078	0.095	0.15	0.28
	HLA-DRA	0.33	***	0.25	***	0.16	0.22
	HLA-DPA1	0.33	***	0.19	***	0.098	0.47
	NRP1	0.51	***	0.39	***	0.3	0.022
	ITGAX	0.31	***	0.21	***	0.23	0.082

BRCA, breast invasive carcinoma; SKCM, skin cutaneous melanoma; UCS, uterine carcinosarcoma. Cor, R value of Spearman’s correlation. *p < 0.01; **p < 0.001; ***p < 0.0001.

### Identification of ACSL4 Related Compounds

**FIGURE 8:**
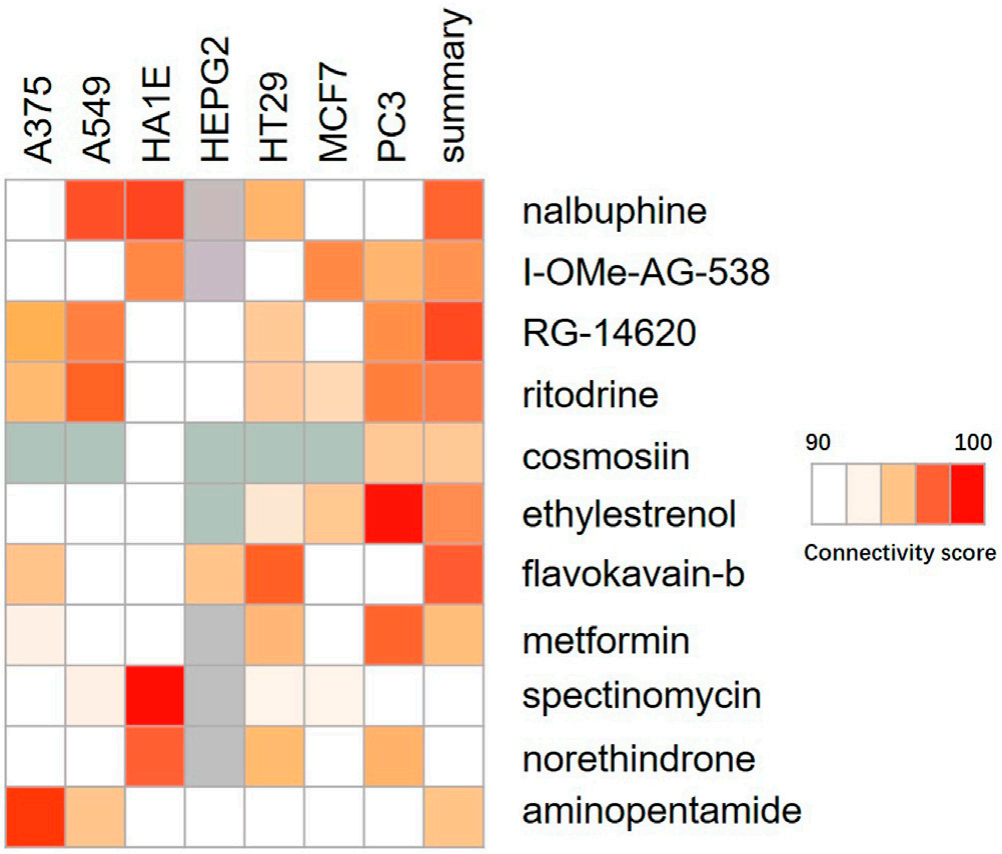
Heat map of compounds interacting with ACSL4. The connectivity score was calculated using kolmogorov-Smirnov two-sided statistics implemented in CMap.

The Connectivity Map was used as a first step in the drug discovery process. By comparing the queried and existing gene signatures, links can be found between gene knockout/overexpression or small molecules with similar or opposite effects (Lamb, 2007). Their related transcriptional effects suggest they confer related physiological effects on the cell. Genes with positive and negative scores have similar and opposite gene characteristics to ACSL4 expression, respectively. Connections were viewed as a heat map ranked by the summary connectivity score. Heat map showed that compounds with high similarity to ACSL4 included inhibitor of Insulin Growth factor 1 receptor protein Tyrosine kinase, nalbuphine opioid receptor agonist, opioid receptor antagonist, EGFR inhibitor, Cytochrome P450 inhibitor and carcinoma cell growth inhibitor. (Figure 8).

## Discussion

Metabolic reprogramming is one of the hallmarks of cancer (Hanahan and Weinberg, 2011). Alterations in lipid metabolism, including catabolism and anabolism, are part of metabolic reprogramming that occurs in tumor cells to promote gene mutations, loss of tumor suppressors, and epigenetic modifications (Currie et al., 2013). Free fatty acids are converted into fatty acyl-coA synthase in an ATP-dependent manner, which causes membrane phospholipid biosynthesis, energy utilization and storage, lipid signaling and other physiological and metabolic processes (Coleman et al., 2000). Dysregulation of fatty acid metabolism leads to excessive lipid biosynthesis and deposition, which promotes the occurrence and development of cancer (Tang et al., 2018). ACSL4 is a key factor involved in metabolism-related diseases. Recent studies have shown that ACSL4 promotes arachidonic acid (AA) and epinephrine lipid peroxidation, participating in ferroptosis-related processes, and overexpression of ACSL4 promotes ferroptosis (Kagan et al., 2017; Cheng et al., 2020). Different from apoptosis, necrosis and autophagy, iron death is an iron-dependent, lipid peroxidation mediated form of cell death (Dixon et al., 2012). Wang W. et al. found that CD8^+^ T cells activated by immunotherapy enhanced the occurrence of lipid peroxidation in tumor cells, which then initiated ferroptosis, further enhancing the anti-tumor effect of immunotherapy (Wang et al., 2019). It suggests that the ferroptosis is involved in T cell mediated cancer immunity. There is a lack of research on the correlation between ferroptosis and immune response.

More and more evidence highlights the important role of ACSL4 in regulating the proliferation and apoptosis of cancer cells. Sung et al. found that ACSL4 was overexpressed in HCC tissues and cells (Sung et al., 2007). Arachidonic acid drives ACSL4 ubiquitination through substrate-induced post-translational regulatory mechanisms (Kan et al., 2014). Recent studies have shown that ACSL4 also increased GLUT1-mediated O-Glcnacylation promotes hepatocellular carcinoma cell growth and survival. In addition, ACSL4 acts as an activator of mTOR signaling, increasing HCC cell growth and inhibiting apoptosis (Wang J. et al., 2020). In breast cancer, ACSL4 promotes breast cancer cell proliferation, invasion and migration through the same mechanism (Orlando et al., 2015). Overexpression of ACSL4 is associated with the development of colon cancer. Apatinib promotes the ferroptosis in CRC cells by targeting ELOVL6 and subsequently regulating ACSL4 expression (Tian et al., 2021). Studies in human glioma have found that ACSL4 expression is down-regulated and iron death is also reduced. Therefore, ACSL4 is proposed to protect glioma cells and play an anti-proliferative role by activating the ferroptosis pathway, and highlights the key role of ACSL4 in regulating ferroptosis in the protection of glioma (Cheng et al., 2020). To analyze ACSL4 expression levels in different cancers, this study analyzed ACSL4 mRNA differential expression in 32 cancer types from Oncomine database and TIMER database compared with normal tissues. The two data sets were consistent: high expression of ACSL4 in colorectal cancer and liver cancer, low expression in breast cancer and central nervous tumor. It’s both elevated and decreased in pancreatic cancer. In addition, the expression was inconsistent in renal cell carcinoma, with high expression in Oncomine and down expression in TIMER, which may be due to differences in data sources and standardized methods of different databases. We also added the analysis results of GEPIA and found that ACSL4 expression was low in BRCA. In addition, the protein expression pattern of ACSL4 was analyzed by UALCAN database, and it was found that ACSL4 was highly expressed in colorectal cancer and low in breast cancer. Further, the prognostic value of ACSL4 in different cancers was evaluated in these databases, and we found that the prognosis of ACSL4 in breast cancer and lung cancer was consistently poor. To be specific, the GEO dataset analyzed by PrognoScan indicates that high expression of ACSL4 is associated with poor prognosis in Colorectal cancer and Brain cancer, and low expression of ACSL4 is associated with poor prognosis in Lung and Breast cancer. Similarly, low expression of ACSL4 in BRCA, LUAD, BLCA, CESC, HNSC, PAAD, STAD and TGCT showed poor prognosis in Kaplan-Meier database. GEPIA explored the poorer prognosis of ACSL4 low expression in ACC, KIRC, LGG, PCPG, and SKCM. These results suggest that ACSL4 has potential prognostic value in breast cancer, lung cancer, skin malignant melanoma and other cancers, and can be used as a biomarker reflecting the prognosis.

Genetic structural variation in the human genome can exist in many forms (Redon et al., 2006). Single nucleotide polymorphism (SNP) is considered to be the main form of structural variation, and CNV is also widely existed, which is associated with the occurrence and development of various cancers (Zhou et al., 2019; Wu et al., 2020; Zhong et al., 2020). There are few studies on the relationship between ACSL4 gene alteration and cancer. In this study, mixed-cancer panel found that ACSL4 had different degrees of genetic changes in 21 cancers, among which ACSL4 was most prone to genetic changes in Endometrial carcinoma, Melanoma and Sarcoma. In the cervical Squamous cell carcinoma, pleural Mesothelioma and Glioblastoma, all the cases were mutation. In Renal Clear cell carcinoma, Hepatocellular carcinoma and well-differentiated Thyroid cancer, Amplification occurred in all cases. Deep deletion occurred only in Seminoma in all cases. In addition, we found that patients with ACSL4 mutations had poor OS and PFS.

In recent years, more and more attention has been paid to the molecular classification of endometrium, which was mainly divided into four subtypes, which were believed to be caused by driver changes, i.e. POLE EDM, TP53 mutation or MMR defect, with significantly different prognosis (León-Castillo et al., 2020). Oxidative stress (OS) played an important role in melanoma metastasis through excessive production of reactive oxygen species (ROS). Other studies have shown that ionizing radiation (IR) not only induced the production of ROS in tumor cells, but also induced ACSL4 expression. ACSL4 preferentially utilized arachidonic acid as a substrate, resulting in increased lipid peroxidation and ferroptosis, thus exerting antitumor activity (Lei G. et al., 2020). This is also consistent with the poor prognosis of the low expression of ACSL4 in melanoma in this study. We further analyzed ACSL4 single nucleotide variation (SNV) using GSCA. The results showed that the most variation was found in UCEC and SKCM, and the prognosis of ACSL4 mutant was worse in SKCM. CNV analysis showed that ACSL4 CNV was positively correlated with ACSL4 expression in 10 cancers, which was consistent with cBioPortal results. The prognosis was worse in patients with copy number deletion in KIPR, CESC, and LAML, and worse in patients with copy number amplification in UCEC. DNA methylation is a common epigenetic modification by linking methyl groups to the C5 site of cytosine (5mC) in the CpG environment (Cao et al., 2019). Hypomethylation has been shown to be widespread in cancer progression (Muhammad et al., 2021; Sugiura et al., 2021). Hypomethylation of ACSL4 in BRCA, BLCA, LIHC, STAD and TCGT was found in the UALCAN database. In GSCA. we found that ACSL4 expression was negatively correlated with methylation levels in 19 cancers. Interestingly, in LIHC, ACSL4 overexpression may be associated with hypomethylation of ACSL4 and a worse prognosis. This is consistent with the correlation between ASLC4 mRNA expression and prognosis in LIHC, that is, high expression of ACSL4 leads to worse prognosis. A previous study also found that hypomethylated CpG sites of ACSL4 were associated with an increased risk of non-alcoholic fatty liver disease (NAFLD) (Zhang et al., 2018). These results suggest that hypomethylation of ACSL4 may be one of the epigenetic regulatory mechanisms in many cancers.

Immune evasion is an important marker of cancer and has long been considered as a basic process of tumor formation and progression (Chen and Mellman, 2017; Lei X. et al., 2020). The prognostic value of immune-infiltrating cells has been extensively studied in various malignancies, but ACSL4 has not been well studied in immuno-oncology (Ge et al., 2021; Yu et al., 2021). A major finding in this study was that ACSL4 expression was associated with levels of immune invasion across multiple cancer types. We found that ACSL4 expression was positively correlated with neutrophils, monocytes and CD4+T cells. As expected, ACSL4 was not consistently associated with levels of infiltration of CD8 T cells, Tfh, Treg, B cells, macrophages, NK, and DC across different cancers. In BRCA and SKCM, ACSL4 was more closely related to the level of immune cell infiltration. In SKCM, ACSL4 expression level was not correlated with tumor purity, suggesting that there was no significant difference in ACSL4 expression level between tumor cells and tumor microenvironment. However, in BRCA, ACSL4 expression levels were significantly negatively correlated with tumor purity, suggesting sustained enrichment of ACSL4 in tumor cells. After adjusting for tumor purity, ACSL4 was significantly positively correlated with the level of immune cell infiltration in SKCM and BRCA. These findings suggest that ACSL4 plays an important role in the recruitment and regulation of immune-infiltrating cells in cancer. Patricia P. Yee et al. found in glioma studies that neutrophils play an anti-tumor role by inducing iron death in tumor cells through myeloperoxidase (MPO) and ACSL4 (Yee et al., 2020). CD4+T cells secrete a variety of cytokines, which directly or indirectly activate other immune cells (such as B cells and CD8 +T cells) and enhance the anti-tumor activity of CTL. The original theory postulated that higher expression of immune cells in the early stages of carcinogenesis contributed to anti-tumor activity (Bindea et al., 2013). Our data analysis confirmed that ACSL4 and higher levels of immune cell infiltration were associated with better survival outcomes in BRCA and SKCM. As cancer progresses, the immune microenvironment may promote cancer cell growth while mediating immunosuppression. Myeloid suppressor cells are immature myeloid cells that also have immunosuppressive effects in the tumor microenvironment. In this study, it was found that cancers with high ACSL4 expression (LICH, COAD, STAD) had high levels of MDSC infiltration, while cancers with low ACSL4 expression (BRCA, KIRC, LUSC, SKCM) had high levels of MDSC infiltration, and had poor prognosis and survival in BRCA and SKCM. The prognosis was consistent with ACSL4 mRNA. Another study analyzed the possible association between tumor mutation load (TMB) prognosis and immune cell infiltration in SKCM. Studies have shown that SNPS are more frequent than insertions or deletions in SKCM, and patients with characteristic mutants in the high TMB group have poor survival outcomes and inhibit immune infiltration levels (Cao et al., 2019).These results suggest that ACSL4 may influence the level of tumor immune cell infiltration and ultimately affect patient survival, providing a reference for immunotherapy.

Considering that ACSL4 is genetically altered in a variety of cancers, we further analyzed whether there is a correlation between ACSL4 gene alteration or epigenetic modification and tumor immune cell infiltration level by using TIMER database. In this study, ACSL4 mutation was significantly positively correlated with CD4+T cells, CD4+T cells, B, DC, macrophages and CAFS infiltration levels in UCEC, while significantly negatively correlated with immune cell infiltration levels in SARC. In UCEC, Mutation mainly occurred, the level of immune cell invasion increased, and the prognosis and survival became worse. In SARC, Amplification and deep deletion mainly occurred, and the level of immune cell invasion decreased. When ACSL4 copy number is abnormal, the infiltration level of CAFs in BRCA and OV is reduced, and the prognosis of ACSL4 mRNA is poor. Although changes in the ACSL4 gene in our data affect immune cell infiltration levels in only a few cancer types, it also suggests that ACSL4 mutations or copy number abnormalities are closely associated with immune cell infiltration and affect prognosis in UCEC, SARC, BRCA and OV. It may be a potential immune and prognostic biomarker. This has not been suggested in previous studies.

In addition, this study further analyzed the association between ACSL4 and immune cell biomarkers in different cancers. It was found that ACSL4 had significant positive correlation with biomarkers of CD4+T cells, TAM, M2 macrophages, monocytes, DC, CAFs and MDSC in BRAC and SKCM. Notably, ACSL4 mRNA expression was low in both cancers and the prognosis was poor. This suggests that ACSL4 may affect prognosis by regulating immune cell recruitment and activation. In addition, ACSL4 was positively correlated with the markers of Exhaust T cells (CTLA4, HAVCR2, LAG3, PDCD1) and Treg cells (CCR8, IL2RA, IL7R). There is increasing evidence that exhaust cells play an inhibitory role in the tumor microenvironment by changing signal cascade and epigenetic metabolism, attenuating effector cytotoxicity, reducing cytokine production, and regulating various inhibitory molecular receptors (Franco et al., 2020; Zeng et al., 2020). Increased infiltration of Exhaust T cells was found in many tumors, and patients with high expression of PD-L1 had better anti-PD1 treatment (Zheng et al., 2017; Guo et al., 2018). These results suggest that ACSL4 may affect the efficacy of immunotherapy and may become a potential predictive biomarker of immunotherapy.

By searching the CMap database, it was found that the compound with high similarity to ACSL4 was mainly inhibitor of Insulin growth factor 1 receptor protein tyrosine kinase. EGFR inhibitor etc. The insulin-like growth factor (IGF) signaling system plays a crucial role in human cancer. IGF-1 receptor (IGF-1R) inhibitors prevent binding of IGF-1 to IGF1R and subsequently inhibit down stream signaling, including PI3K/Akt pathway, exerting antitumor activity and being potential targets for cancer treatment (Pollard and Daniel, 2019). In this study, we speculate that the mechanism of ACSL4 in cancer is closely related to the IGF signaling pathway. These highly similar compounds may contribute to the treatment of cancers with elevated ACSL4 expression, including colorectal cancer and liver cancer. However, CMap model is based on microarray data, and the accuracy of prediction may be limited. A large number of experimental studies are still needed to develop and verify the efficacy of the drug.

In this study, the expression and prognostic value of ACSL4 in different cancers were analyzed using multiple online public databases. Individual cancer results may be inconsistent due to differences in data collection and standardization across different databases. Therefore, further *in vivo* and *in vitro* experiments are needed to realize the mechanisms of ACSL4 in different cancer types at the cellular and molecular level. This is what the Bioinformatics Research Institute lacks. Although ACSL4 expression was found to be associated with immune cell infiltration and patient survival, it has not been demonstrated that ACSL4 affects patient survival through immune infiltration. ACSL4 is not only a sensitive monitor of ferroptosis, but also an important contributor of ferroptosis. The relationship between acSL4-mediated ferroptosis and immune infiltration and the clinical response to immunotherapy are also important topics for future research.

## Conclusion

This study analyzed the expression of ACSL4 in generalized carcinoma, and for the first time analyzed the genetic and epigenetic changes of ACSL4 in different cancers, as well as its impact on survival and prognosis. In addition, ACSL4 mediates the level of immune cell infiltration in BRCA and SKCM, providing new ideas for personalized cancer immunotherapy. ACSL4 is expected to be a potential prognostic and prognostic immunotherapy biomarker.

## Data Availability

The datasets presented in this study can be found in online repositories. The names of the repository/repositories and accession number(s) can be found in the article/Supplementary Material.
