# Supplementary figures and images for: Genetic Alteration, Prognostic and Immunological Role of Acyl-CoA Synthetase Long-Chain Family Member 4 in a Pan-Cancer Analysis
Source: Front Genet. 2022 Jan 20;13:812674. doi: 10.3389/fgene.2022.812674 (PMC8811308; doi:10.3389/fgene.2022.812674)

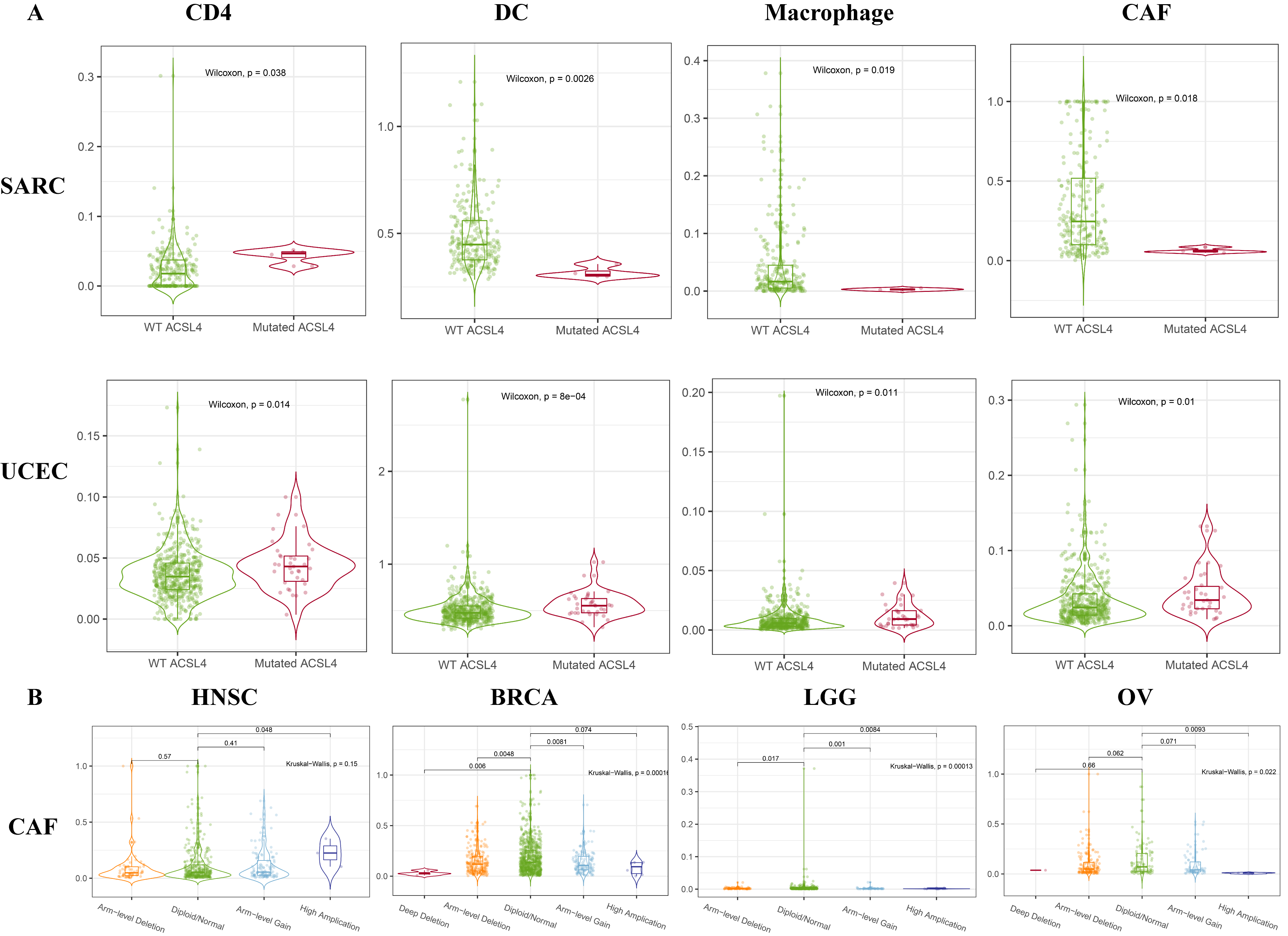

Supplement: Supplementary file 3 [file Image6.TIF]

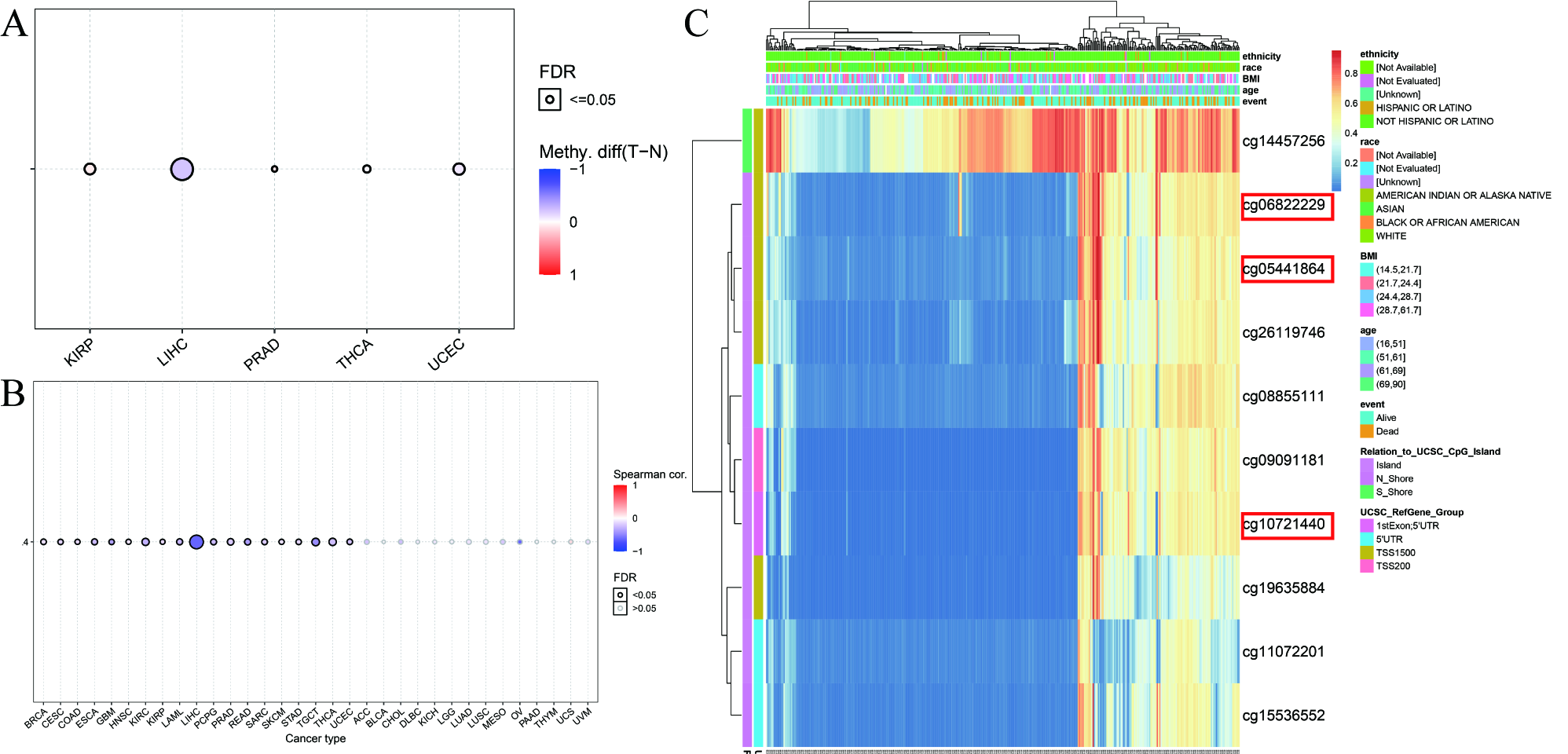

Supplement: Supplementary file 4 [file Image3.TIF]

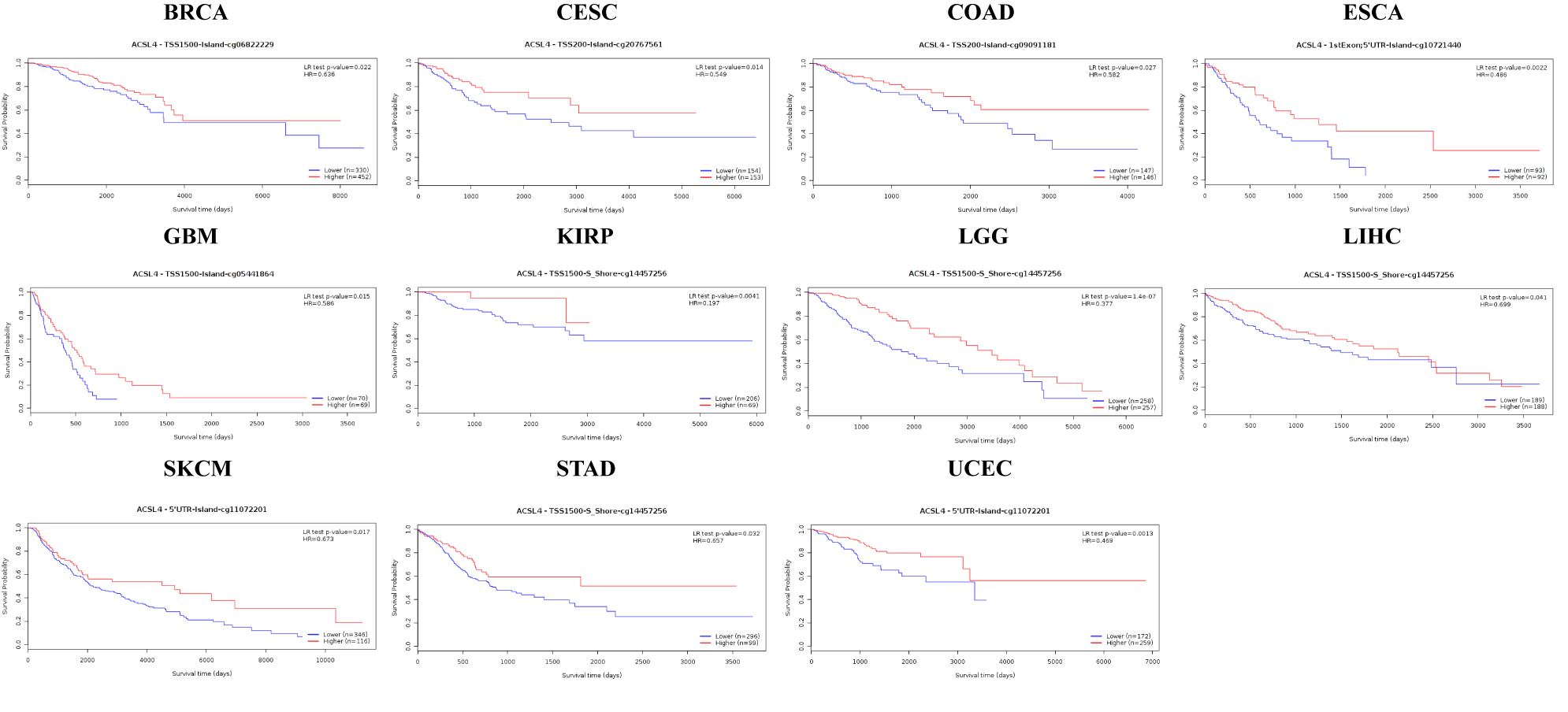

Supplement: Supplementary file 5 [file Image4.TIF]

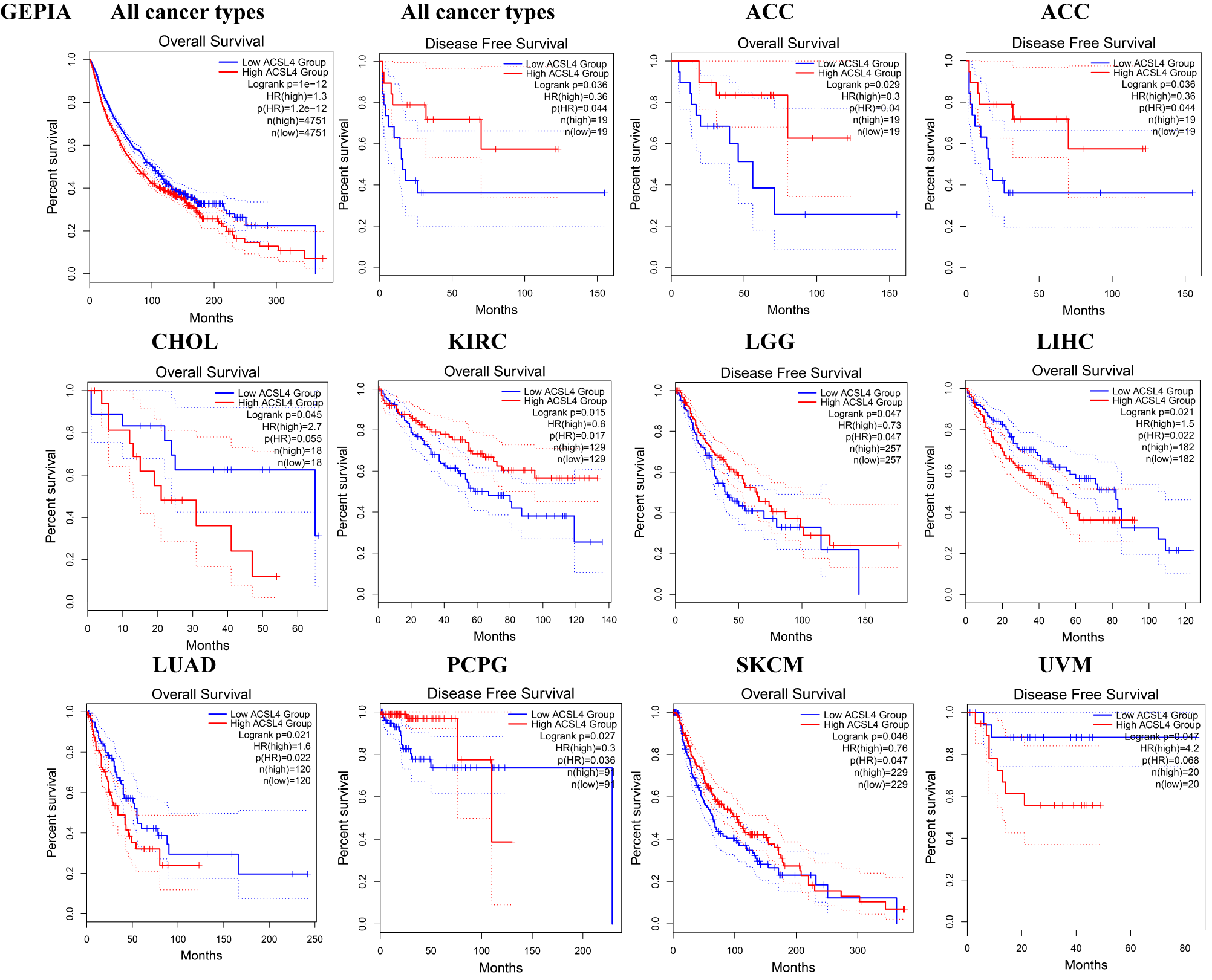

Supplement: Supplementary file 6 [file Image2.TIF]

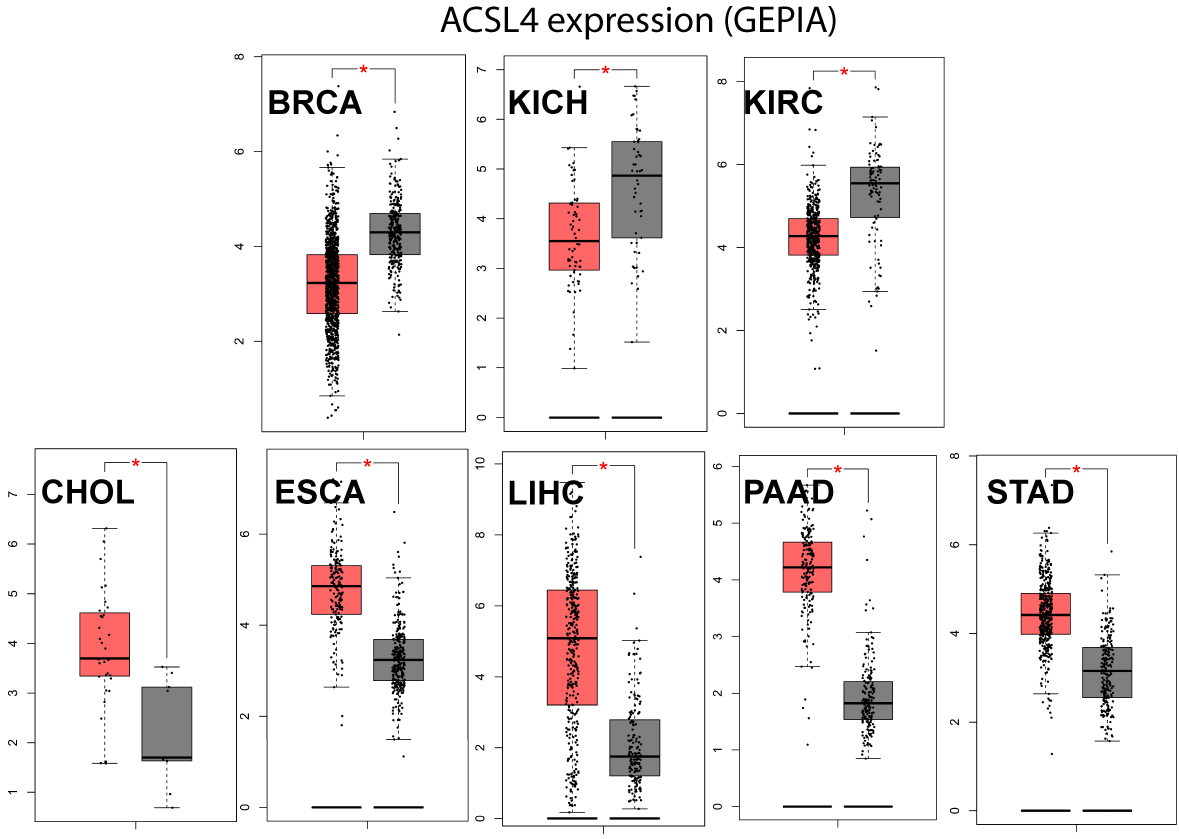

Supplement: Supplementary file 7 [file Image1.TIF]

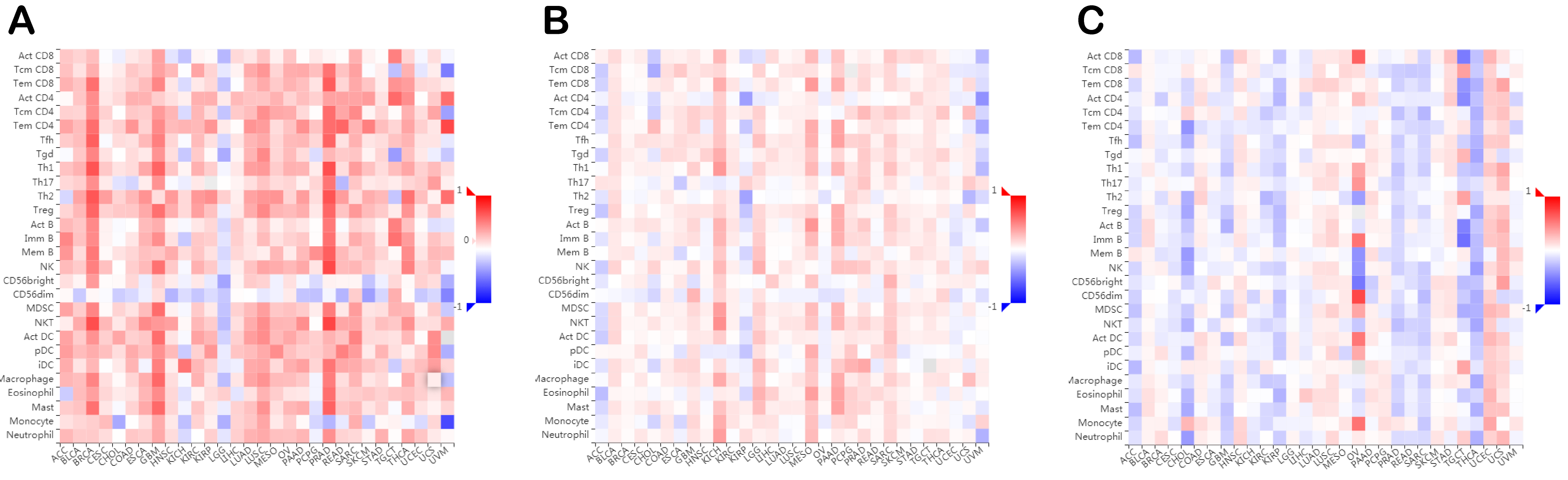

Supplement: Supplementary file 10 [file Image5.TIF]
